# *Streptomyces* sp. Strain PBR11, a Forest-Derived Soil *Actinomycetia* with Antimicrobial Potential

**DOI:** 10.1128/spectrum.03489-22

**Published:** 2023-01-31

**Authors:** Rajkumari Mazumdar, Partha Pratim Dutta, Juri Saikia, Jagat Chandra Borah, Debajit Thakur

**Affiliations:** a Life Sciences Division, Institute of Advanced Study in Science and Technology, Guwahati, India; b Department of Molecular Biology and Biotechnology, Cotton University, Guwahati, India; c Department of Biotechnology, Gauhati University, Guwahati, India; d Assam Down Town University, Guwahati, India; North-West University

**Keywords:** *Actinobacteria*, *Actinomycetia*, *Actinomycetota*, antimicrobial, *Streptomyces* sp., MDR, MIC, TLC, flash chromatography, LC-MS/MS, cytotoxicity, MTT assay

## Abstract

The *Actinomycetia* isolate PBR11 was isolated from the forest rhizosphere soil of Pobitora Wildlife Sanctuary (PWS), Assam, India. The isolate was identified as *Streptomyces* sp. with 92.91% sequence similarity to their closest type strain, Streptomyces atrovirens NRRL B-16357 DQ026672. The strain demonstrated significant antimicrobial activity against 19 test pathogens, including multidrug-resistant (MDR) clinical isolates and dermatophytes. Phenol, 2,5-bis(1,1-dimethylethyl), is the major chemical compound detected by gas chromatography-mass spectrometry in the ethyl acetate extract of PBR11 (EtAc-PBR11). The presence of the PKS type II gene (type II polyketide synthases) and chitinase gene suggested that it has been involved in the production of antimicrobial compounds. Metabolic profiling of the EtAc-PBR11 was performed by thin-layer chromatography and flash chromatography resulted in the extraction of two bioactive fractions, namely, PBR11Fr-1 and PBR11Fr-2. Liquid chromatography-tandem mass spectrometry analysis of both the fractions demonstrated the presence of significant antimicrobial compounds, including ethambutol. This is the first report on the detection of antituberculosis drug in the bioactive fractions of *Streptomyces* sp. PBR11. EtAc-PBR11 and PBR11Fr-1 showed the lowest MIC values (>0.097 and >0.048 μg/mL, respectively) against Candida albicans MTCC 227, whereas they showed the highest MIC values (>0.390 and >0.195 μg/mL, respectively) against Escherichia coli ATCC BAA-2469. The effects of PBR11Fr-1 were investigated on the pathogens by using a scanning electron microscope. The results indicated major morphological alterations in the cytoplasmic membrane. PBR11Fr-1 exhibited low cytotoxicity on normal hepatocyte cell line (CC-1) and the percent cell viability started to decline as the concentration increased from 50 μg/mL (87.07% ± 3.22%) to 100 μg/mL (81.26% ± 2.99%).

**IMPORTANCE** Novel antibiotic breakthroughs are urgently required to combat antimicrobial resistance. *Actinomycetia* are the principal producers of antibiotics. The present study demonstrated the broad-spectrum antimicrobial potential of an *Actinomycetia* strain *Streptomyces* sp. strain PBR11 isolated from the PWS of Assam, India, which represents diverse, poorly screened habitats for novel microorganisms. The strain displayed 92.4% sequence similarity with genes of the closest type strain, indicating that the strain may represent a novel taxon within the phylum *Actinomycetota*. The metabolomics studies of EtAc-PBR11 revealed structurally diverse antimicrobial agents, including the detection of the antituberculosis drug ethambutol, in the bioactive fraction of *Streptomyces* sp. PBR11 for the first time. The PBR11 strain also yielded positive results for the antibiotic synthesis gene and the chitinase gene, both of which are responsible for broad-spectrum antimicrobial activity. This suggests that the untouched forest ecosystems have a tremendous potential to harbor potent actinomycetia for future drug discovery.

## INTRODUCTION

Infectious diseases are a major health care burden around the globe. Antimicrobial treatment for rising multidrug-resistant bacterial and fungal infections is severely limited, but numerous causes are responsible for the emergence of drug-resistant strains (1). Most pathogenic strains have developed resistance to the standard antibiotics due to the inappropriate or misuse of antibiotics over an extended period. However, antibiotic resistance frequently renders existing drugs ineffective, highlighting the critical need for novel antibiotics (2). Therefore, new antibiotics and antimicrobial agents are urgently required to efficiently target the infectious microorganisms that cause life-threatening infections and address the emergence of multidrug-resistant pathogens.

One good attempt in this regard is to mine the microbial biodiversity of untapped regions, which are expected to be inhabited by an uncharted and novel class of actinomycetia. Drug-resistant pathogen strains are emerging faster than new drugs and antibiotics are being discovered. As a result, many researchers and pharmaceutical companies have been actively engaged in isolating and screening actinomycetia from various unscreened habitats to develop novel antibiotics. *Streptomyces* is one of the largest bacterial genera and the principal producer of antibiotics. It belongs to the order *Streptomycetales*, family and class *Actinomycetia* (formerly *Actinobacteria*) (3). Most actinomycetia in soil that are potential drug sources are uncultivable, making novel antibiotic discovery more challenging. Northeast India is a part of the Indo-Burma biodiversity hot spots (4), and screening of actinomycetia from this region deserves special attention to investigate the potential of the diverse microflora. These areas are still understudied and represent diverse ecosystems, as well as the least studied area for isolating actinomycetia with potent extracellular antimicrobial metabolites. The *Actinomycetota* represent the most widely distributed and one of the largest bacterial phyla that exist in a wide variety of aquatic and terrestrial habitats (5). Exploration of actinomycetia for the discovery of novel genera producing secondary metabolites has been going on for decades (6, 7). The *Actinomycetia* class continues to provide new biomolecules to the biotechnology and pharmaceutical industries. Most of these species have a unique mycelial life cycle, forming aerial and substrate mycelium with distinct coloration and sporulation (8, 9). Members of this class are the principal producers of biosynthetic gene clusters of polyketides (type I and type II) and nonribosomal peptides, which are the most efficient producers of diverse groups of novel secondary metabolites. They demonstrate diverse bioactivities, including antimicrobial, anticancer, antiviral, anticholesterol, immune-suppressing, and anti-inflammatory activity, thus attracting many pharmaceutical, chemical, and industrial sectors (10). Actinomycetia are well known for producing almost 80% of the world’s antibiotics, mainly from the genera *Streptomyces* and *Micromonospora* (11). The genus *Streptomyces* is a prolific producer of bioactive molecules and a rich source of novel secondary metabolites with biotechnological applications (12, 13). They are the chief sources of natural products and have a history of developing many biologically active compounds, such as tetracycline, ivermectin, streptomycin, nystatin, etc. Moreover, several new antimicrobial agents (e.g., GE2270A, deoxyactagardine B, and actinonin) and anticancer compounds (e.g., salinosporamide A, sungsanpin, diketopiperazines, and marthiapeptide A) (14–16) are under evaluation. Thiocoraline (17) is a newly isolated depsipeptide from *Micromonospora*. It exhibits strong cytotoxicity against P-388, A-549, and MEL cell lines, as well as antibacterial activity against Gram-positive bacteria. This compound binds to supercoiled DNA and prevents RNA synthesis. Cyclomarins A to C (18) are cyclic peptides produced by *Streptomyces* spp. They exhibit antiviral and anti-inflammatory properties. These breakthroughs have led to the development of improved antimicrobial agents that are believed to benefit humanity (19). Almost 80 years of research on the class *Actinomycetia* has discovered many important drugs that continue to save many human lives worldwide. In addition, various metabolic engineering strategies and sophisticated methods can help in enhancing the value of these bacteria in terms of productivity or creating diverse biologically active products (20). The discovery of novel microbes and products derived from poorly explored areas such as Northeast India, China, Australia, Antarctica, and Jordan suggests that careful investigation of new habitats may continue to be beneficial (21–24). A successful approach for finding new pharmacological leads or chemical scaffolds is investigating novel taxa from untapped sources (25, 26). Actinomycetia have the potential to produce a variety of structurally diverse secondary metabolites for drug development; however, only a small portion of these bacteria have been cultured. The present investigation aimed to isolate *Streptomyces* from the poorly explored protected forest ecosystem of Assam with the capability of producing secondary metabolites with high antimicrobial action. We emphasize here the exploration of *Streptomyces* from the uninvestigated forest ecosystem of Assam, India, for natural products in drug discovery.

## RESULTS

### Morphological identification of *Streptomyces* sp. PBR11 strain.

**FIG 1:**
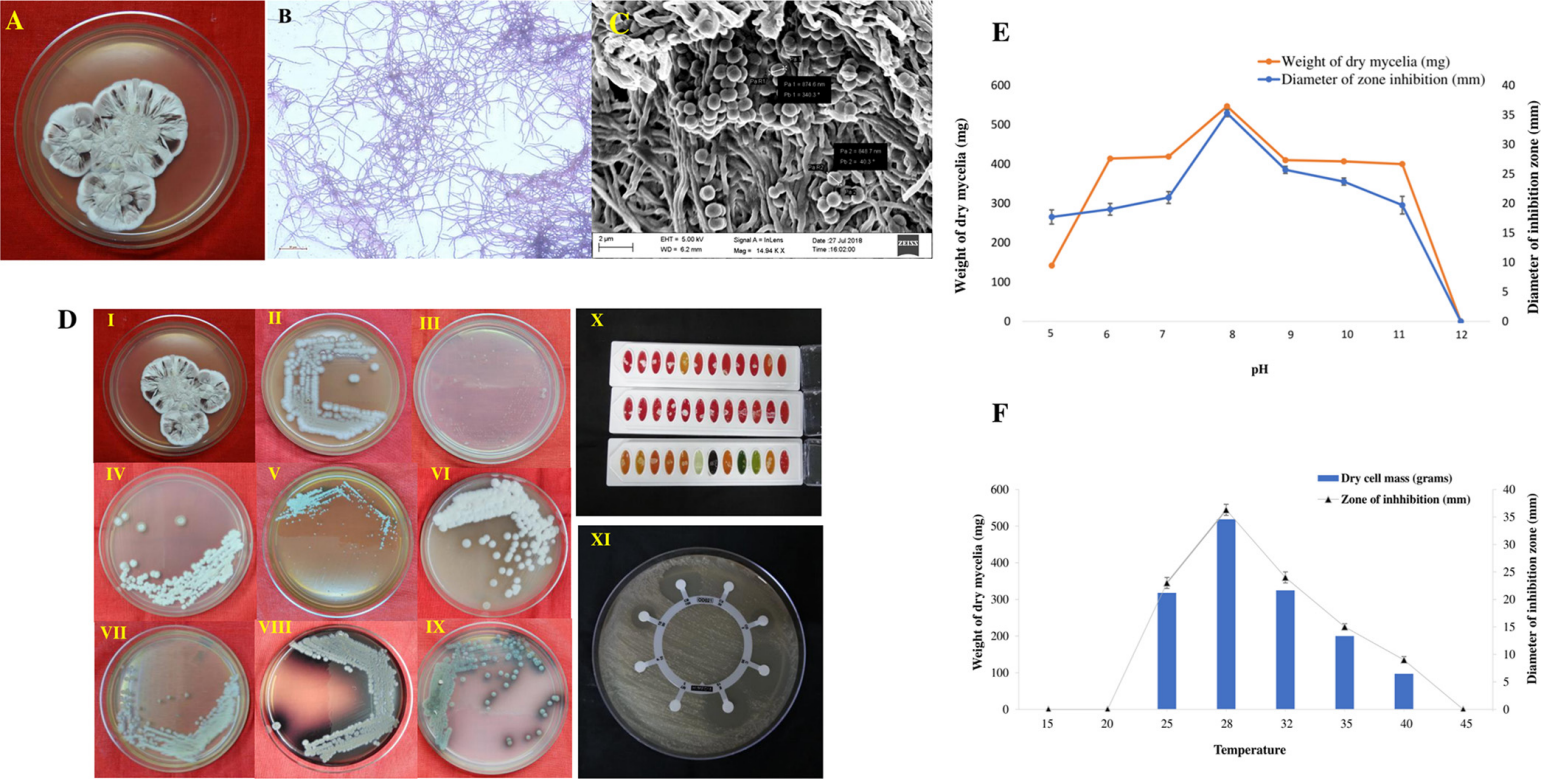
(A) Pure culture plate of *Streptomyces* sp. PBR11 on GLM agar medium. (B) Light microscopic view PBR11 strain. (C) SEM view PBR11 strain showing spore chain morphology. (D) PBR11 strain grown in different culture media for cultural characterization. (I) GLM medium. (II) *Streptomyces* agar. (III) Actinomycetes isolation agar. (IV) Mueller-Hinton agar. (V) Sabouraud dextrose agar. (VI) ISP3. (VII) ISP4. (VIII) ISP6. (IX) ISP7. (X) Carbohydrate utilization test using HiCarbo kit. (XI) *In vitro* antibiotic sensitivity test of PBR 11 strain following disc diffusion method against eight standard antibiotics. (E) Effect of pH on growth and antimicrobial activity assessed in terms of diameter of inhibition zone and dry cell by PBR11 strain. (F) Effect of temperature on growth and antimicrobial activity assessed in terms of diameter of inhibition zone and dry cell by the PBR11 strain.

*Streptomyces* sp. strain PBR11, which is aerobic and filamentous in nature, was isolated from the forest rhizosphere soil of Pobitora Wildlife Sanctuary (PWS), Assam, India. The PBR11 strain was morphologically identified by observing the colony morphology. The aerial mycelium was light purple, whereas the vegetative substrate mycelium produced a light brown color (Fig. 1A). The pure culture of the PBR11 strain grown in GLM agar (described in Materials and Methods) produced an earthy odor.

### Antimicrobial potential of PBR11 strain.

**FIG 2:**
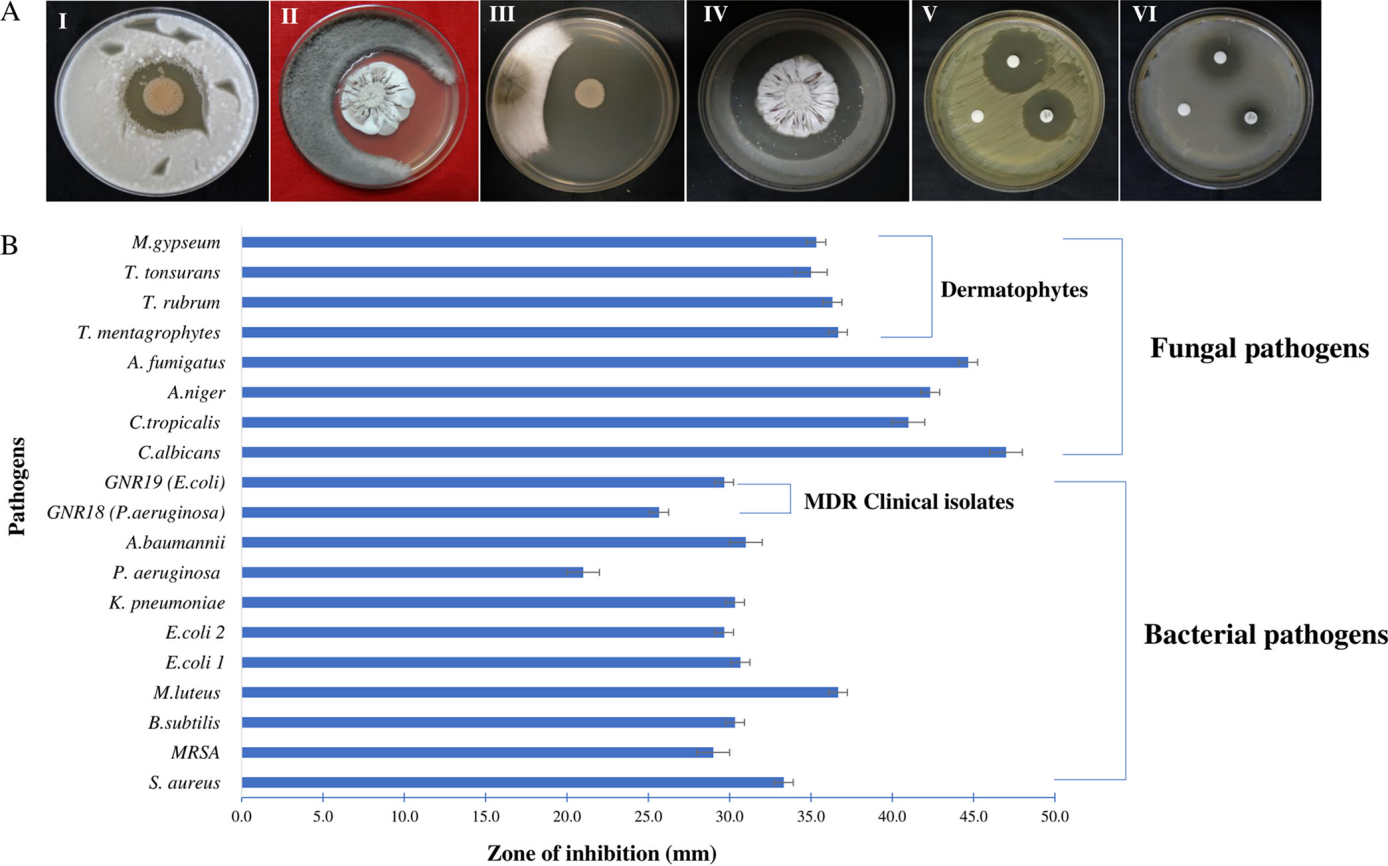
(A) *In vitro* antimicrobial activity of *Streptomyces* sp. PBR11 as determined by spot inoculation (I to IV) and disc diffusion (V and VI) against test pathogens. (I) Trichophyton rubrum MTCC 8477. (II) Aspergillus fumigatus MTCC 1811. (III) Trichophyton mentagrophytes MTCC 8476. (IV) Candida albicans MTCC 227. (V) Micrococcus luteus MTCC 1538 (VI) Escherichia coli MTCC 739. (B) Graph representing the antimicrobial activity of *Streptomyces* sp. PBR11. Each bar denotes the mean of three independent replicates, and the error bar indicates the standard error of the mean of the three replicates. (E. coli 1, E. coli ATCC BAA-2469; E. coli 2, E. coli MTCC 739).

The PBR11 strain demonstrated promising antimicrobial activity during bioactive screening for the production of extracellular secondary metabolites in GLM agar medium by the spot inoculation technique. The PBR11 strain exhibited maximum antifungal activity against Candida albicans MTCC 227 (47.0 ± 1.0), followed by Aspergillus *fumigates* MTCC 1811 (44.7 ± 0.6), Aspergillus niger MTCC 282 (42.3 ± 0.6 mm), and Trichophyton mentagrophytes MTCC 8476 (36.7 ± 1.0 mm). The maximum inhibition zone among bacterial test pathogens was observed in Micrococcus luteus MTCC 1538 (36.7 ± 0.6 mm), followed by Staphylococcus aureus MTCC 96 (32.3 ± 0.6 mm) and Acinetobacter baumannii ATCC BAA-1705 (31.0 ± 1.0 mm). The complete antimicrobial profiles of PBR11 strain are shown in Table 1. The antimicrobial activity of the ethyl acetate extract of PBR11 (EtAc-PBR11), along with the controls (10% dimethyl sulfoxide [DMSO]; negative control and antibiotics; positive control) against Micrococcus luteus MTCC 1538 and GNR19 (Escherichia coli) are shown in Fig. 2.

**TABLE 1:** Antimicrobial potential of *Streptomyces* sp. PBR11 strain^*a*^

Sr. no.	Test pathogen	Mean zone of inhibition (mm) ± SD
Bacteria		
1	Staphylococcus aureus (MTCC 96)^*c*^	33.3 ± 0.6
2	MRSA (ATCC 43300)^*b*^	29.0 ± 1.0
3	Bacillus subtilis (MTCC 441)^*c*^	30.3 ± 0.6
4	Micrococcus luteus (MTCC 1538)^*c*^	36.7 ± 0.6
5	Escherichia coli (MTCC 739)^*b*^	29.7 ± 0.6
6	Escherichia coli (ATCC BAA-2469)^*c*^	30.7 ± 0.6
7	Klebsiella pneumoniae (MTCC 3384)^*c*^	30.3 ± 0.6
8	Pseudomonas aeruginosa (MTCC 741)^*d*^	21.0 ± 1.0
9	Acinetobacter baumannii (ATCC BAA-1705)^*c*^	31.0 ± 1.0
MDR clinical isolates		
10	GNR18 (Pseudomonas aeruginosa)^*b*^	25.7 ± 0.6
11	GNR19 (Escherichia coli)^*b*^	29.7 ± 0.6
Yeast		
12	Candida albicans (MTCC 227)^*d*^	47.0 ± 1.0
13	Candida tropicalis (MTCC 184)^*d*^	41.0 ± 1.0
Fungus		
14	Aspergillus niger (MTCC 282)^*d*^	42.3 ± 0.6
15	Aspergillus fumigatus (MTCC 1811)^*d*^	44.7 ± 0.6
16	Trichophyton mentagrophytes (MTCC 8476)^*c*^	36.7 ± 1.0
17	Trichophyton rubrum (MTCC 8477)^*c*^	36.3 ± 0.6
18	Trichophyton tonsurans (MTCC 8475)^*c*^	35.0 ± 1.0
19	Microsporum gypseum (MTCC 8469)^*c*^	35.3 ± 0.6

a The production of extracellular secondary metabolites was screened by spot inoculation method in GLM agar medium. The zone of inhibition was determined after 24 to 48 h of incubation at 37°C for bacteria and 28°C for yeasts and fungi. The values for the zone of inhibition are given as means ± standard deviations of the mean (*n* = 3). Sr. no., serial number.

b Zone of inhibition, 20mm – 30mm: Pseudomonas aeruginosa (MTCC 741), GNR18 (Pseudomonas aeruginosa), MRSA (ATCC 43300), Escherichia coli (MTCC 739), GNR19 (Escherichia coli).

c Zone of inhibition, 30.1mm – 40mm: Bacillus subtilis (MTCC 441), Klebsiella pneumoniae (MTCC 3384), Escherichia coli (ATCC-BAA 2469), Acinetobacter baumannii (ATCC BAA-1705), Staphylococcus aureus (MTCC 96), Trichophyton tonsurans (MTCC 8475), *Microsporum gypseum* (MTCC 8469), Trichophyton rubrum (MTCC 8477), Trichophyton mentagrophytes (MTCC 8476), Micrococcus luteus (MTCC 1538).

d Zone of inhibition, 40.1mm–50mm: Candida tropicalis (MTCC 184), Aspergillus niger (MTCC 282), Aspergillus fumigatus (MTCC 1811), Candida albicans (MTCC 227).

### Biochemical characterization and optimization of cultural conditions on growth and antimicrobial production.

The aerial mycelia are long and branched, as confirmed by scanning electron microscopy (SEM) analysis. Spore chains are rectiflexibiles with 10 or more spores per chain. Each chain has oval-shaped spores that range in size from 848.7 to 874.6 nm in diameter (Fig. 1C). Table 2 shows the cultural traits of the PBR11 strain in different culture mediums, where the strain grew well on all media except actinomycetes isolation agar medium (Fig. 1D). The strain was found to grow at between pH 5 and pH 11. The maximum growth and highest secondary metabolite production were obtained at a pH of 8 (Fig. 1E and Table 3). However, poor growth and low antimicrobial production were detected at pH levels above pH 11 and below pH 4. The optimum temperature for maximum growth and highest antibiotic production was 28°C. The highest production of secondary metabolites and best growth was observed on the third day under shake-flask conditions at 28°C. To evaluate the potentiality of PBR11 stain for novel antibiotics, eight standard antibiotics were used for the antibiotic susceptibility test; of these, the strain showed resistance to nitrofurantoin (300 μg), ampicillin (10 μg), and co-trimoxazole (25 μg). The complete biochemical characteristics of strain PBR11 and its antibiotic susceptibility profile are shown in Table 4.

**TABLE 2:** Cultural characteristics of *Streptomyces* sp. PBR11 on different culture media^*a*^

Culture medium	Aerial mycelium color	Substrate mycelium color	Diffusible pigment	Growth
GLM medium	Light purple	Light brown	−	+++
Streptomycetes agar	White	Cream	−	+++
Actinomycetes isolation agar	Cream white	Cream	−	+
Mueller-Hinton agar	Cream	Cream	−	+++
Sabouraud dextrose agar	White	Cream	−	++
ISP3	White	Cream	−	+++
ISP4	White	Cream	−	+++
ISP6	Dark brown	Light brown	Dark black	+++
ISP7	Brown	Light brown	Black	+++

a +++, good growth; ++, moderate growth; +, poor growth; −, no diffusible pigment produced. The strain grew well (+++) on several media, including GLM medium, streptomycetes agar, Mueller-Hinton agar, ISP3, ISP4, ISP6, and ISP7. It showed moderate growth (++) on Sabouraud dextrose agar but poor growth (+) on actinomycetes isolation agar. Diffusible pigments were observed in ISP6 and ISP7 afyer incubation at 28°C for 5 days; pH 8. ISP, International Streptomyces Project.

**TABLE 3:** Effect of pH on growth and antimicrobial activity assessed in terms of inhibition zone diameter and dry cell mass by *Streptomyces* sp. PBR11^*a*^

pH	Dry cell mass (g)	Mean zone of inhibition (mm) ± SD	Growth
3	−	−	−
4	−	−	−
5	0.142	17.7 ± 1.2	+
6	0.414	19.0 ± 1.0	+++
7	0.419	21.0 ± 1.0	+++
8	0.547	35.3 ± 0.6	+++
9	0410	25.7 ± 0.6	++
10	0.407	23.7 ± 0.6	++
11	0.400	19.7 ± 1.5	++
12	−	−	−

a Test pathogen: Candida albicans MTCC 227. The strain was inoculated in GLM broth at 28°C for 8 days at various pH ranges, from acidic to alkaline (pH;3 to 12), to assess growth and antimicrobial activity in terms of dry cell mass and inhibition zone diameter. The growth was recorded at various pH levels as follows: +++, good growth; ++, moderate growth; +, poor growth; or −, no growth.

**TABLE 4:** Biochemical characteristics of *Streptomyces* sp. PBR11

Utilization of carbon source or antibiotic sensitivity	Response or sensitivity^*a*^
Carbon source	
Trehalose	+
Lactose	−
Xylose	−
Maltose	+
Fructose	−
Dextrose	+
Galactose	−
Raffinose	−
Melibiose	−
Sucrose	+
l-Arabinose	+
Mannose	+
Insulin	+
Sodium gluconate	−
Glycerol	+
Salicin	+
Dulcitol	−
Inositol	−
Sorbitol	−
Mannitol	+
Adonital	+
Arbitol	−
Erythritol	−
l-Methyl-d-glucoside	−
Rhamnose	+
Cellubiose	+
Melezitose	+
l-Methyl-d-mannoside	+
Xylitol	+
ONPG	+
Esculin hydrolysis	+
d-Arabinose	+
Citrate utilization	−
Malonate utilization	−
Sorbose	+
Control	−
Antibiotic sensitivity (μg/disc)	
Nitrofurantoin (300)	R
Norfloxacin (10)	S
Ampicillin (10)	R
Cefotaxime (30)	S
Cephalothin (30)	S
Co-trimoxazole (25)	R
Gentamycin (10)	S
Nalidixic acid (30)	S

a +, Positive for test; −, negative for test; S, sensitivity; R, resistant.

### Gas chromatography-mass spectrometry analysis.

**FIG 3:**
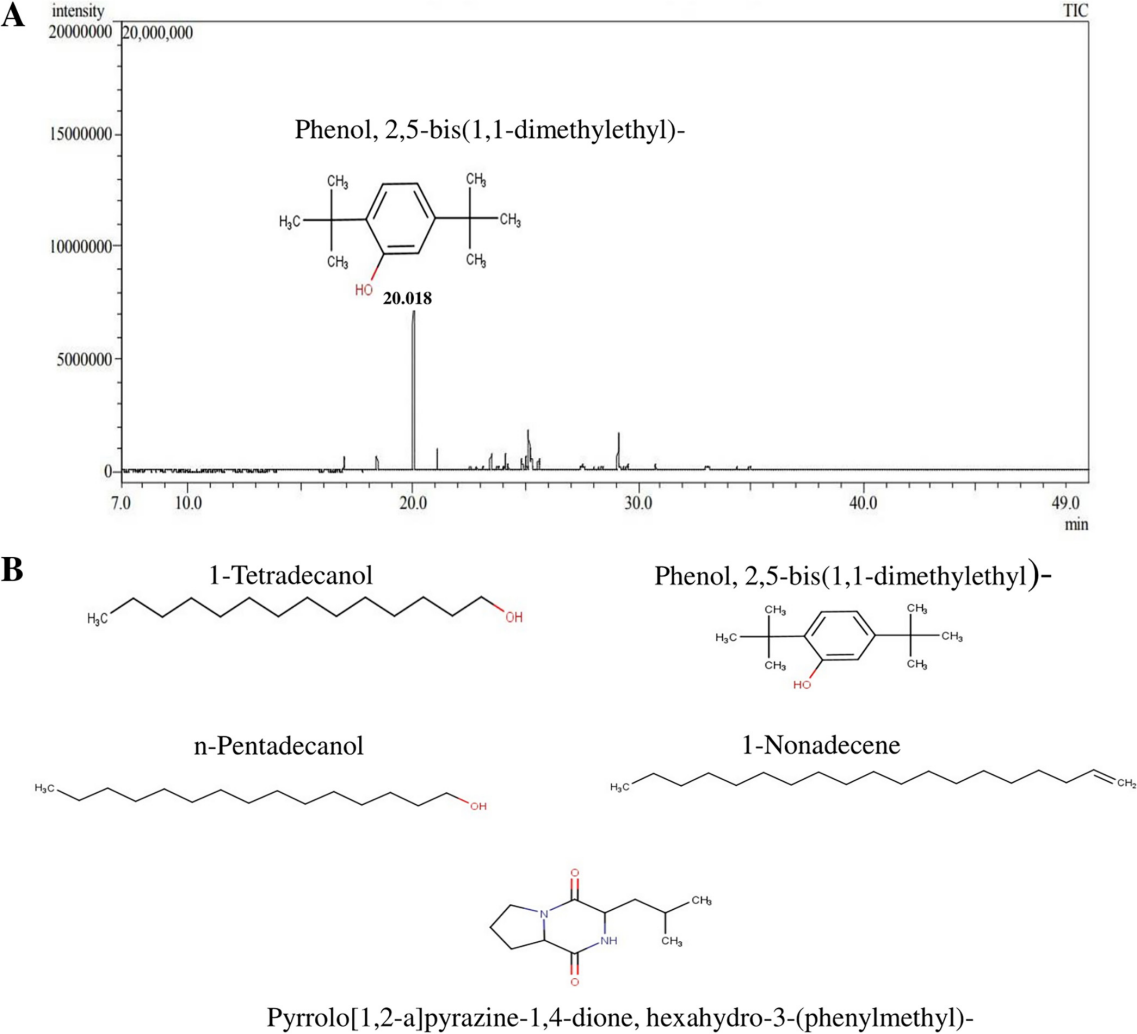
(A) Graph illustrating the GC-MS analysis of an EtAc extract of PBR11 showing major phenolic compounds. (B) Structure of chemical compounds detected in EtAc-PBR11 by GC-MS analysis.

The analysis of bioactive constituents present in the crude extract EtAc-PBR11 was evaluated by gas chromatography-mass spectrometry (GC-MS). Five major chemical compounds were detected based on molecular weight and their retention times by comparing their mass spectra with the National Institute of Standards and Technology (NIST) database. Most of the detected compounds are known to have antibacterial, antifungal, anticancer, antioxidant, and antituberculosis activities. The results are shown in Table 5, and the structures of the identified compounds and GC-MS chromatogram graph of EtAc-PBR11 are shown in Fig. 3. The peak area of the detected compound indicates the quantity of compounds in the extract.

**TABLE 5:** Chemical compounds detected in EtAc-PBR11 by GC-MS analysis^*a*^

Compound name	RT	MW (g/mol)	Area (%)	Nature of compound	Activity	Reference(s)
1-Tetradecanol	18.386	214.39	2.44	Alcohol	Antibacterial activity	48
Phenol, 2,5-bis(1,1-dimethylethyl)-	20.018	206.32	33.03	Phenol	Antimicrobial activity	24
*n*-Pentadecanol	21.061	228.41	4.03	Alcohol	Antibacterial activity	49, 50
1-Nonadecene	23.41	266.50	2.86	Alkene	Antituberculosis, anticancer, antioxidant, antifungal, antimicrobial	51 – 53
Pyrrolo[1,2-*a*]pyrazine-1,4-dione, hexahydro-3-(phenylmethyl)-	29.118	244.29	10.42	Aromatic organic compound	Antimicrobial activity	54, 55

a RT, retention time; MW, molecular weight of compounds.

### Molecular characterization and phylogenetic analysis of PBR 11 strain.

**FIG 4:**
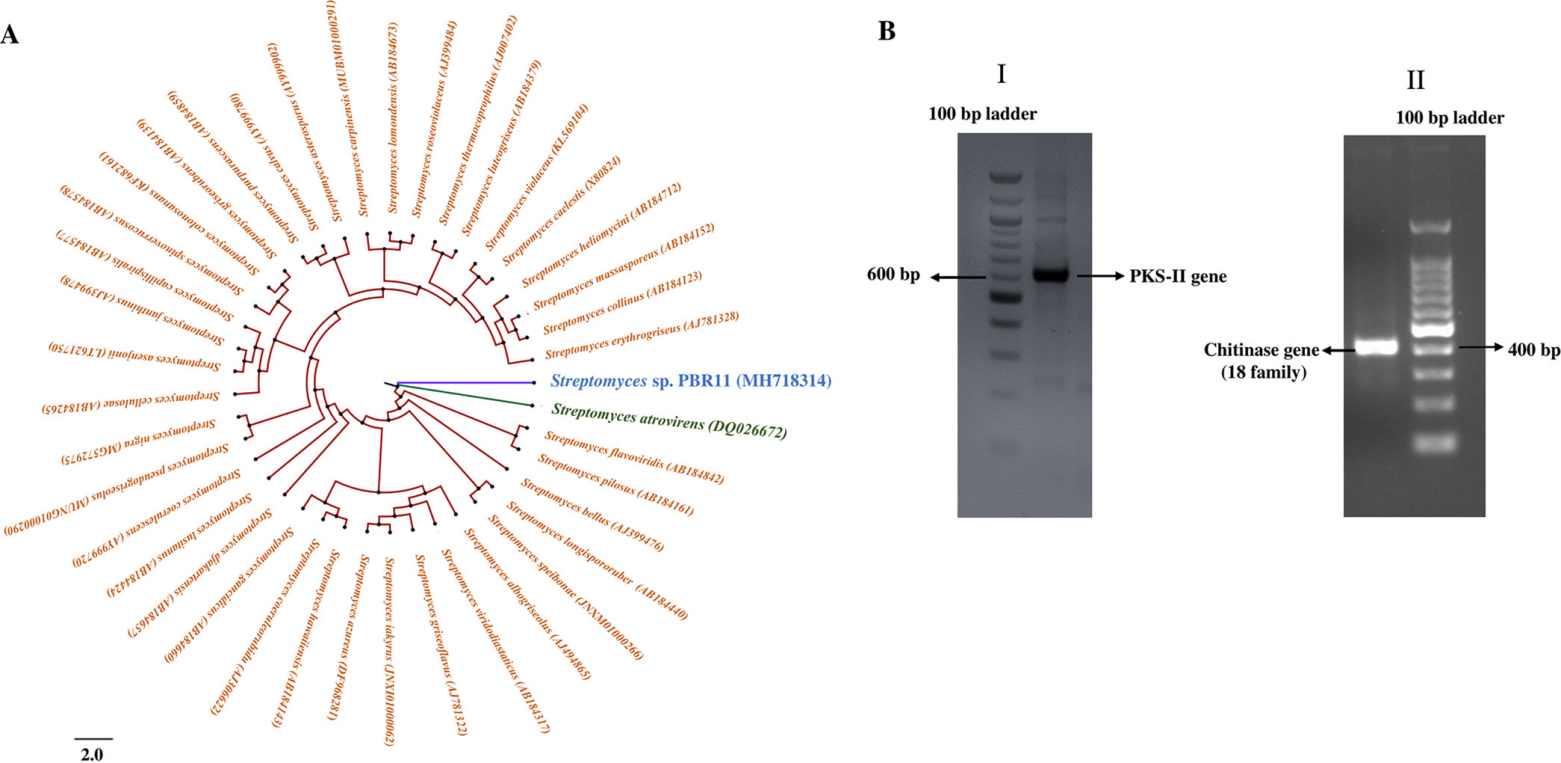
(A) Phylogenetic tree of *Streptomyces* sp. PBR11 and the closest *Streptomyces* species showing phylogenetic relationships based on the 16S rRNA gene sequences. Phylogenetic analysis was performed by the maximum-likelihood method using MEGA X with 1,000 bootstrap steps. (B) Agarose gel electrophoresis of PCR-amplified products of PBR11. (I) Amplification of chitinase gene of glycoside hydrolase family 18 using GA1F and GA1R specific primers; (II). Amplification of polyketide synthases type II gene (PKS-II) using degenerate primers KSαF and KSβR.

The partial 16S rRNA gene sequence of the PBR11 strain was deposited into the NCBI GenBank database under the accession number MH718314. The gene size for the partial sequences is 1,370 bp. The strain showed the highest sequence similarity with Streptomyces atrovirens strain NRRL B-16357 (DQ026672). Based on a neighbor-joining approach, the phylogenetic tree also displayed its highest similarity to Streptomyces atrovirens (92.91%) (Fig. 4A). The phenotypic data and molecular identification indicated that the PBR11 strain belonged to the genus *Streptomyces*, and thus the strain was designated as *Streptomyces* sp. PBR11.

### Detection and investigation of the biosynthetic gene PKS type II and chitinase gene for the prediction of chemical classes.

*Streptomyces* sp. PBR11 showed positive results against both biosynthetic genes clusters PKS-II and for chitinase 18 Glycosyl Hydrolase family gene (GH18) (Fig. 4B). The partial sequences of the PKS-II gene and chitinase gene were deposited in GenBank under accession numbers ON911582 and ON911583, respectively. The amino acid sequence of the PKS type II gene showed close similarity to beta-ketoacyl synthase family protein and shared 99.42% similarity with their closest match *Streptomyces* sp. (WP_004922256) at the amino acid level. The predicted protein structure displayed the KS domain, which is similar to the structure of actinorhodin polyketide putative beta-ketoacyl synthase of Streptomyces coelicolor A3(2) (70.9% identity, PDB ID 1tqy) responsible for polyketide antibiotic biosynthesis. The predicted protein product showed maximum similarity to macrolide antibiotic megalomicin. The translated protein sequence of the chitinase GH18 gene showed the highest similarity to GH18 protein with 98.18% similarity to their closest match Streptomyces katrae (WP_045948892). The predicted protein structures were similar to the crystal structure of chitinase 40 of the thermophilic actinomycetia Streptomyces thermoviolaceus (70.9% identity, PDB ID 4w5u) involved in chitin degradation (Table 6).

**TABLE 6:**
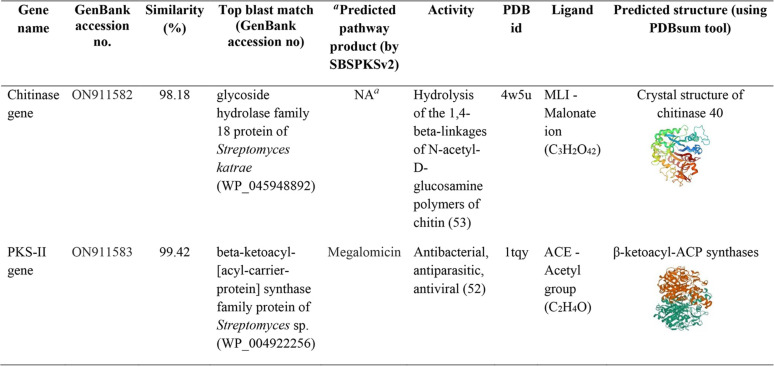
Amino acid sequence similarities of the chitinase gene and biosynthetic gene PKS-II of *Streptomyces* sp. PBR11 and their predicted gene product by SBSPKSv2 and PDBsum tool

a NA, not applicable.

### Metabolic profiling analysis of PBR11Fr-1 and PBR11Fr-2 by TLC and LC-MS/MS.

**FIG 5:**
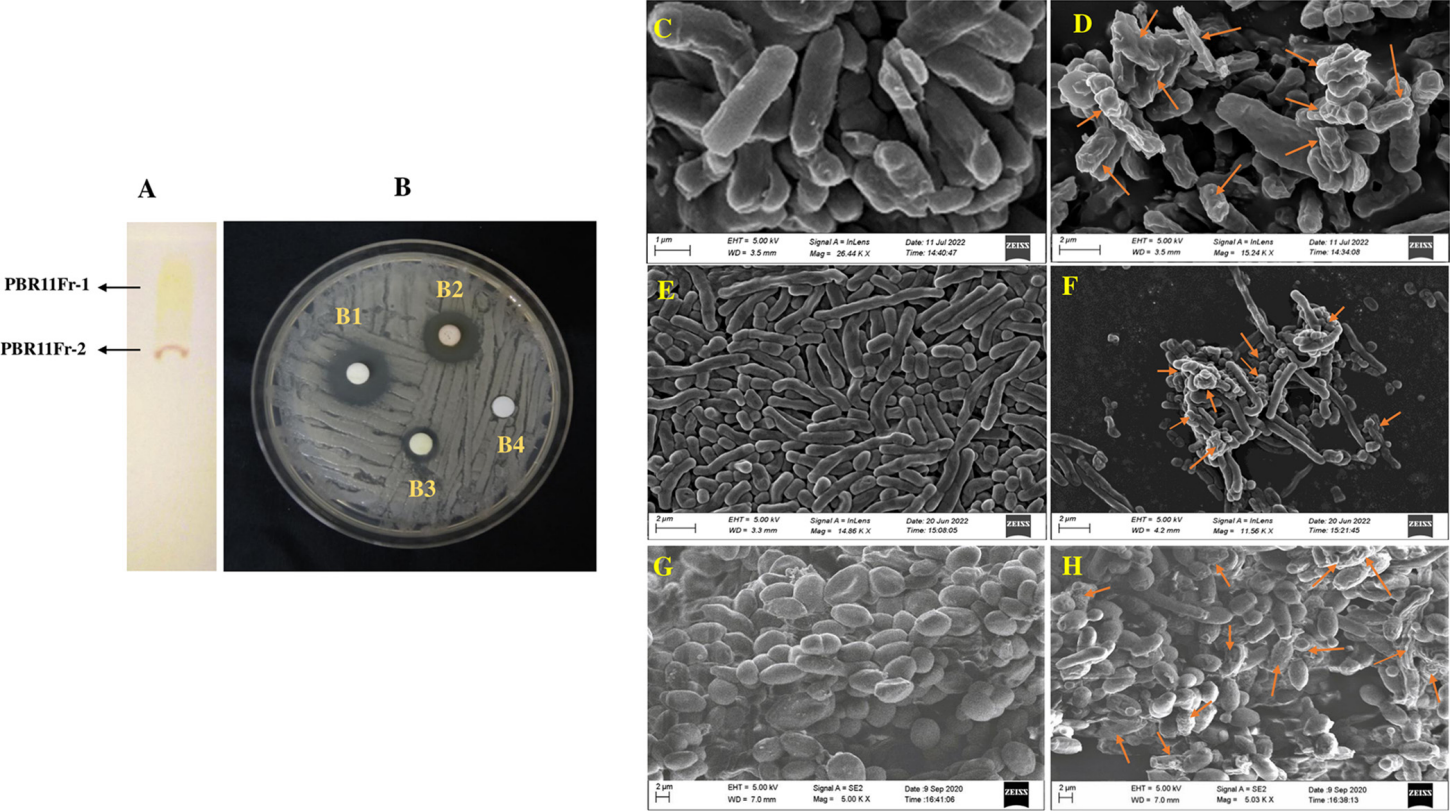
(A) TLC of isolated bioactive fractions PBR11Fr-1 and PBR11Fr-2. (B) *In vitro* antimicrobial activity of bioactive fractions against C. albicans MTCC 227 in PDA (potato dextrose agar) media: B1, 20 μg of PBR11Fr-1; B2, 20 μg of PBR11Fr-2; B3, 20 μg of amphotericin B (positive control); and B4, 10% DMSO (negative control). SEM images showing the effects of PBR11Fr-1 on E. coli ATCC BAA-2469, A. baumannii ATCC BAA-1705, and C. albicans MTCC 227 cells. (C, E, and G) Control cells. (D, F, and H) Treated cells. Arrows denote ruptured cells after treatment.

**FIG 6:**
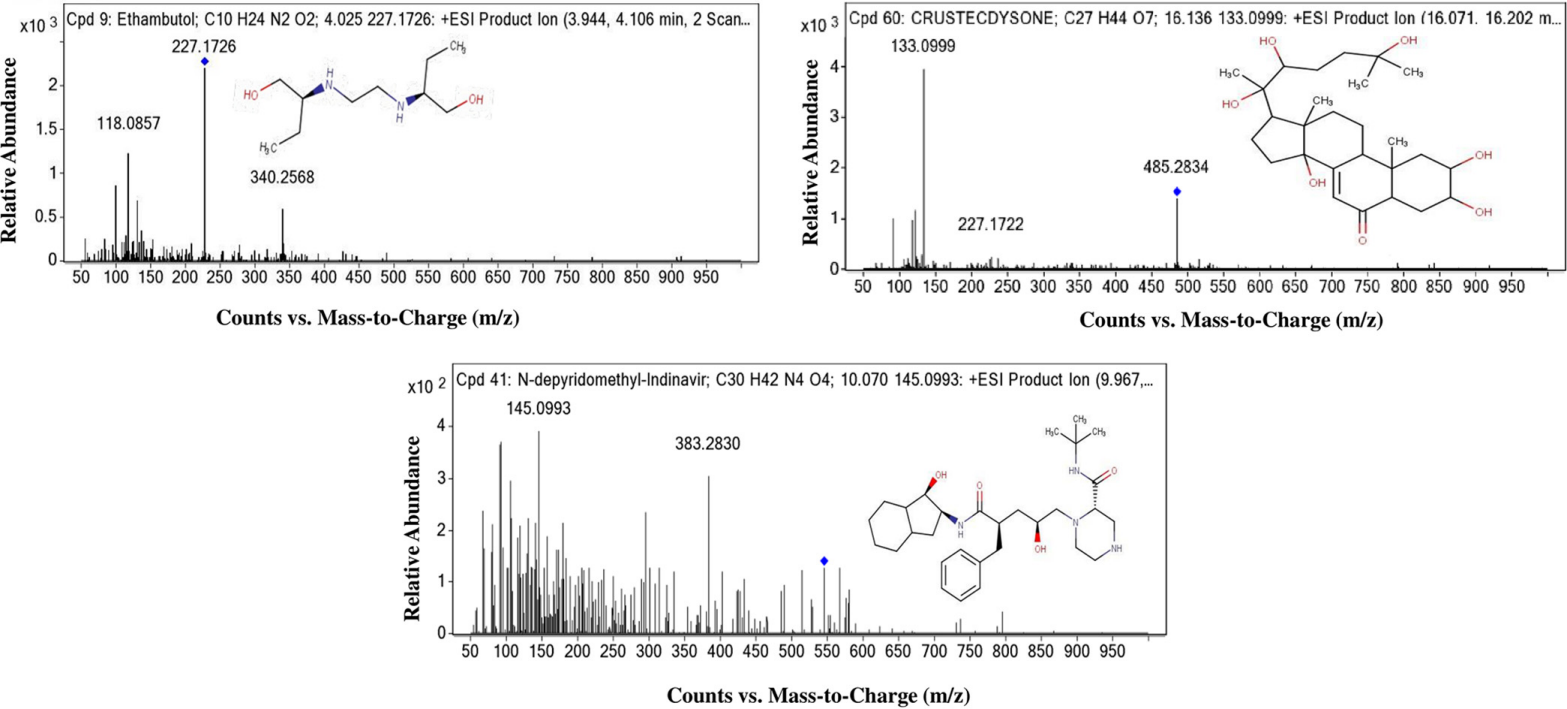
LC-MS/MS spectra of ethambutol, *N*-depyridomethyl-indinavir, and crustecdysone (20-hydroxyecdysone) with their structures detected in both bioactive fractions PBR11Fr-1 and PBR11Fr-2.

A crude extract of *Streptomyces* sp. PBR11 was subjected to analytical thin-layer chromatography (TLC) to determine the ideal solvent systems for the compound separation. Among the various solvent systems used, the best resolution of crude extract was obtained with a chloroform-methanol (30:70 [vol/vol]) system. By repeated chromatographic methods, most bioactive fractions PBR11Fr-1 and PBR11Fr-2 were isolated from the *Streptomyces* sp. PBR11 strain. TLC of the isolated fractions, along with the bioactivity of the fraction against Candida albicans MTCC 227, was shown in Fig. 5A and B. Chemical profiling of both the bioactive fractions showed that they produce a diverse group of bioactive compounds belonging to different chemical classes. The bioactive fraction PBR11Fr-1 contains a total of 19 major compounds, 12 of which—*viz.* arecoline, ethambutol,10-nitro-9*Z*,12*Z*-octadecadienoic acid, campestanol, *N*-depyridomethyl-indinavir, SM(d18:0/0:0), ethoxyquin, phendimetrazine, colforsin, 10-deoxymethymycin, anandamide (20:2, *n*-6), and miltefosine—exhibited diverse biological activities. Ten different compounds were detected in the PBR11Fr-2 fraction. Eight of the compounds are known to have biological activities, including ethambutol, diethylcarbamazine, acetylsalicylic acid (aspirin), 2-propyl-9*Z*-octadecenoic acid, *N*-depyridomethyl-indinavir, oleandrin, 12,14-pentacosadiynoic acid, and crustecdysone (20-hydroxyecdysone). These compounds are known to produce antituberculosis, anti-inflammatory, antidiabetic, anticancer, antioxidant, antibacterial activity, antiretroviral, and antiparasitic activities, respectively. The liquid chromatography-tandem mass spectrometry (LC-MS/MS) findings for ethambutol, *N*-depyridomethyl-indinavir, and crustecdysone (20-hydroxyecdysone), along with their structures, are shown in Fig. 6. The identified compounds, elemental compositions, masses, retention times, and biological activities for both PBR11Fr-1 and PBR11Fr-2 fractions are presented in Table 7.

**TABLE 7:** Chemical compounds present in PBR11Fr-1 and PBR11Fr-2 fractions determined by LC-MS/MS

Fraction	RT	Chemical formula	Mass	*m/z*	Activity (reference)
PBR11Fr-1					
11-Amino-undecanoic acid	1.07	C_11_H_23_NO_2_	201.17	202.18	
Arecoline	1.08	C_8_H_13_NO_2_	155.10	155.09	Anthelmintic activity (78, 79)
Ethambutol	4.02	C_10_H_24_N_2_O_2_	204.18	227.17	Antituberculosis activity (76, 77)
10-Nitro,9*Z*,12*Z*-octadecadienoic acid	7.70	C_18_H_31_NO_4_	325.22	308.22	Anti-inflammatory (80)
Campestanol	9.02	C_28_H_50_O	402.38	407.36	Hypocholesterolemic effect (83)
*N*-Depyridomethyl-Indinavir	9.96	C_30_H_42_N_4_O_4_	522.32	545.31	Potent HIV-1 inhibitor (71, 72)
SM(d18:0/0:0)	9.98	C_23_H_52_N_2_O_5_P	467.36	472.33	Cell signaling (84)
Dinorpromazine	10.06	C_15_H_16_N_2_S	256.10	279.09	
Ethoxyquin	10.46	C_14_H_19_NO	217.15	218.16	Antioxidant, anticarcinogen (85, 86)
Pyrrhoxanthin	10.85	C_39_H_48_O_6_	612.34	595.34	
Phendimetrazine	11.56	C_12_H_17_NO	191.13	174.12	Anorexigenic agent (87)
Colforsin	11.90	C_22_H_34_O_7_	410.23	415.21	Anti-inflammatory effects, apoptosis (81, 82)
d-Pantetheine 4′-phosphate	14.19	C_11_H_23_N_2_O_7_PS	358.10	341.09	
3-Hydroxytetradecanedioic acid	14.65	C_14_H_26_O_5_	274.18	279.15	
10-Deoxymethymycin	15.09	C_25_H_43_NO_6_	453.30	454.31	Antibacterial (88)
Anandamide (20:2, *n*-6)	16.59	C_22_H_41_NO_2_	351.31	352.32	Fatty acid neurotransmitter (89)
Crustecdysone (20-hydroxyecdysone)	16.89	C_27_H_44_O_7_	480.30	485.29	Antioxidant, anti-inflammatory, immunomodulatory, antiobesity and antidiabetic activities, neuroprotective and hepatoprotective agent, antiparasitic activity, inhibits caspase activity and induces autophagy (73–75)
Miltefosine	17.08	C_21_H_46_NO_4_P	407.31	390.31	Antileishmanial (90)
*N*-(2-Hydroxyethyl) icosanamide	20.51	C_22_H_45_NO_2_	355.34	338.34	
PBR11Fr-2					
11-Amino-undecanoic acid	1.103	C_11_H_23_NO_2_	201.17	202.18	
Ethambutol	3.843	C_10_H_24_N_2_O_2_	204.18	227.17	Antituberculosis activity (76, 77)
Diethylcarbamazine	4.953	C_10_H_21_N_3_O	199.17	222.16	Anti-inflammatory effects (91)
5,11-Eicosadienoic acid	7.032	C_20_H_36_O_2_	308.29	313.25	
Acetylsalicylic acid (aspirin)	7.737	CH_3_COOC_6_H_4_COOH	180.04	163.06	Nonsteroidal anti-inflammatory drug (92)
2-Propyl-9*Z*-octadecenoic acid	7.74	C_21_H_40_O_2_	324.29	329.28	Antiplasmodial activity (93)
*N*-Depyridomethyl-Indinavir	9.971	C_30_H_42_N_4_O_4_	522.32	545.31	Potent HIV-1 protease inhibitor (71, 72)
Oleandrin	10.667	C_32_H_48_O_9_	576.33	577.33	Anticancer and novel antiviral activity (95)
12,14-Pentacosadiynoic acid	10.691	C_25_H_42_O_2_	374.31	397.30	
Crustecdysone (20-hydroxyecdysone)	16.202	C_27_H_44_O_7_	480.30	485.29	Antioxidant, anti-inflammatory, immunomodulatory, antiobesity, and antidiabetic activities; neuroprotective and hepatoprotective agent; antiparasitic activity; inhibits caspase activity and induces autophagy (73–75)

### MICs of EtAc-PBR11 and PBR11Fr-1.

The broth dilution technique was used to study the MIC of EtAc-PBR11 and bioactive fraction PBR11Fr-1 ranging from 50 to 0.047 μg/mL against three test pathogens:s Candida albicans MTCC 227, Escherichia coli ATCC BAA-2469, and Acinetobacter baumannii ATCC BAA-1705. PBR11Fr-1 exhibited lowest MIC against C. albicans MTCC 227 (>0.048 μg/mL), followed by A. baumannii (>0.097 μg/mL) and E. coli (>0.195 μg/mL). DMSO (10%) was used as a control and had no activity on the test pathogens (Table 8).

**TABLE 8:** EtAc-PBR11 and bioactive fraction PBR11Fr-1 MICs as determined by broth dilution^*a*^

Test pathogen	MIC (μg/mL)			
	EtAc-PBR11	PBR11Fr-1	Levofloxacin	Amp B
C. albicans (MTCC 227)	≥0.097	≥0.048	NA	≥0.97
E. coli ATCC BAA-2469	≥0.390	≥0. 195	≥8	NA
A. baumannii ATCC BAA-1705	≥0. 195	≥0.097	≥8	NA

a Twofold serial dilutions of the extracts (working solution, 50 to 0.048 μg/mL) were prepared for MIC tests. The media used were nutrient agar for bacteria and potato dextrose broth for C. albicans. NA, not applicable; Amp B, amphotericin B.

### Morphological effect of PBR11Fr-1 on the test pathogens.

SEM analysis of C. albicans MTCC 227, E. coli ATCC BAA-2469, and A. baumannii ATCC BAA-1705 cells after treatment with 1× MIC PBR11Fr-1 revealed significant morphological changes compared to the control cells (Fig. 5C to H). The cytoplasmic membrane structures of treated cells displayed deformities, which led to cell shrinkage and the loss of cell integrity. The cell surfaces of the nontreated cells (control) were intact and retained their original cell structures and morphologies with an intact cell surface.

### Cytotoxic activity of bioactive fraction PBR11Fr-1.

**FIG 7:**
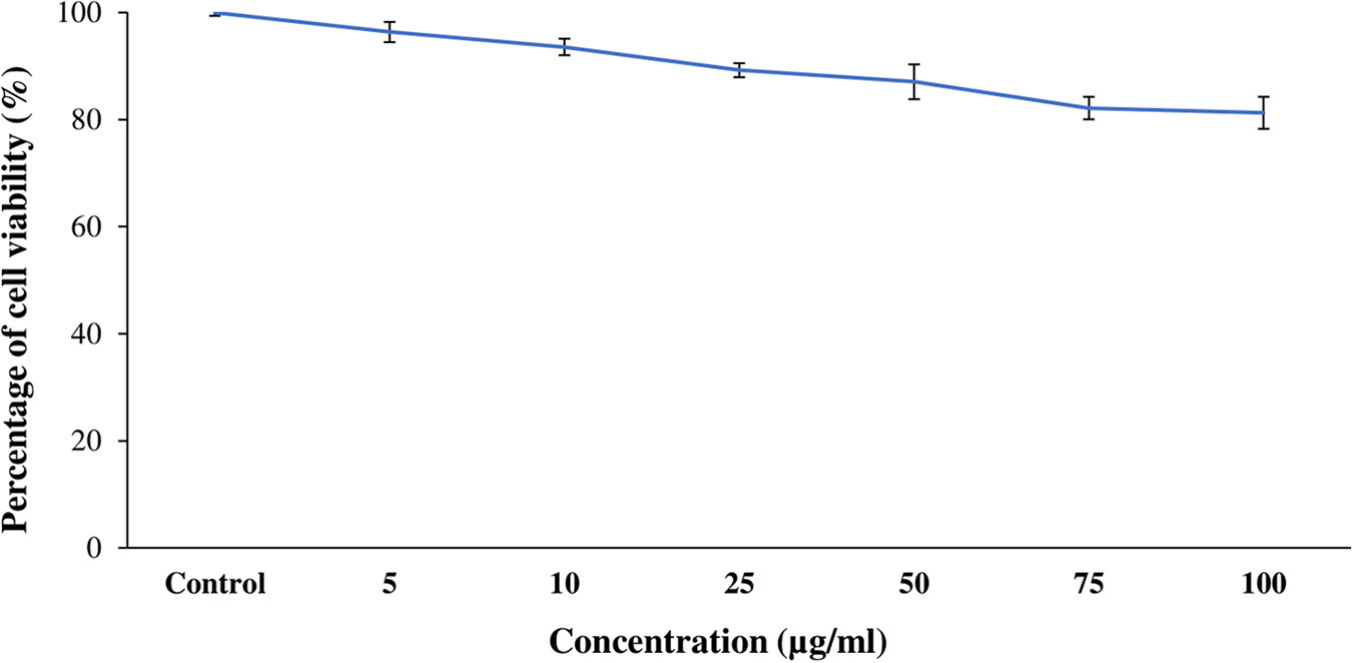
Cytotoxicity of bioactive fraction PBR11Fr-1 isolated from *Streptomyces* sp. PBR11 against normal liver cells (CC-1).

The toxicity of the PBR11Fr-1 on normal liver cell line CC-1 was evaluated at different concentrations. The highest percentage of cell viability was recorded at 5 μg/mL (96.37% ± 1.89%). The cell viability started declining with the increase in the concentration from 50 μg/mL (87.07% ± 3.22%) to 100 μg/mL (81.26% ± 2.99%). The PBR11Fr-1 fraction exhibited a low cytotoxic effect in a normal cell line (Fig. 7).

## DISCUSSION

Infectious diseases are the leading cause of death worldwide, impacting health care and socioeconomic development. Furthermore, the rapid development of drug resistance to currently available antimicrobial agents and adverse side effects from prolonged use is a major public health concern. Therefore, the development of novel antimicrobial drugs is urgently required. Actinomycetia are the most prolific producers of natural bioactive compounds, especially antibiotics and many other bioactive compounds with diverse biological properties (4, 27). Isolation of microorganisms from untouched ecosystems leads to the possibility of discovering novel microbial products, particularly when it pertains to the north-eastern region of India (28). The majority of the forest area of this region is a part Indo-Burma biodiversity hot spots, and there is an excellent possibility that these forest ecosystems may contain various novel microbes capable of producing diverse secondary metabolites. PWS is located on the south bank of the Brahmaputra River (26°12″ to 26°16″N and 91°58″ to 92°05″E), about 50 kilometers east from Guwahati, the state capital of Assam, India. The climate of the PWS is subtropical monsoon. The rainy season, which lasts from May to September, has an average rainfall of 2,000 mm. This period is very humid and warm. The maximum average temperature is 25°C, and the humidity is >95% (29). The entire region is covered by the Brahmaputra flood plains. Because it is low-lying, it experiences yearly flooding. The soil is mostly made up of river alluvium, and its color ranges from reddish-brown to reddish yellow. The soil type is fertile clayey loam with silt (28). Although it is a small area, it is biologically diverse and is located in one of the world’s megabiodiversity hot spots. Using serial dilution technique, a total of 65 morphologically different putative actinomycetia were isolated and purified from the soil samples of the unique niches of PWS. The serial dilution technique is a well-established microbiological technique that has been used successfully to improve culturability and facilitate the separation of molecularly detectable bacteria and archaea from freshwater, marine, and soil systems (30–34). Schoenborn and his team hypothesized in 2004 that liquid serial dilution should also enable the isolation of microbes from poorly investigated bacterial families from an unsaturated soil (35).

*Streptomyces* sp. PBR11 was isolated from the rhizosphere soil of *Cynodon dactylon*, commonly known as Bermuda grass. Based on the results of *in vitro* antimicrobial bioassay, of 65 actinomycetia isolates, the most potent isolate, PBR11, was selected for further studies.

The aerial mycelia of the PBR11 strain are long and branching. Spore chains are rectiflexibiles, and the spore shape is globose. PWS of Assam, India, is the least-explored area for the isolation of microorganisms particularly actinomycetia, and only a few studies on their antimicrobial biosynthetic capacity have been published (28). It has been previously reported that an actinomycetia strain, *Nocardia* sp. PB-52, with broad spectrum bioactivity was isolated from the PWS (28). Our present work represents the first report on the isolation of a *Streptomyces* genus from this unscreened habitat that demonstrated broad-spectrum action against 19 different test pathogens, including multidrug-resistant clinical isolates and dermatophytes. Fungi are eukaryotic microorganisms, and they utilize mechanisms similar to those of higher animals for synthesizing proteins and nucleic acids. Therefore, it is exceedingly challenging to identify antifungal agents that specifically inhibit fungal metabolism without being toxic to people (36). There is evidence that the dermatophytes, which cause skin infections (dermatophytosis) in humans, have developed resistance to some antimycotic medications (37, 38). The PBR11 strain exhibited strong antifungal activity against all the tested fungal pathogens responsible for causing fungal infections. It has been already reported that *Streptomyces* species remains a prominent source of antimicrobial agents with low cytotoxic effects (39, 40).

The production medium and its growing parameters needed to be optimized to increase the production of antimicrobial metabolites. *Streptomyces* sp. strain PBR11 is a mesophilic bacterium, and its highest antibiotic production capability was recorded at 28°C and pH 8. GLM medium was found to be the most effective growth medium for evaluating the antagonistic activity of the strain while it showed the least growth on actinomycetes isolation agar. The strain showed diffusible pigments on ISP6 and ISP7 media. The ideal incubation period for the synthesis of antimicrobial metabolites was noted on the third day of growth. Our findings slightly deviate from previous reports. Earlier, a report stated that *Streptomyces* sp. isolated from pristine habitats produced maximum antimicrobial metabolites after the fifth day of culture at a pH of 7 (41). Similarly, the actinomycetia strain *Streptomyces* sp. VITSVK5 isolated from the marine sediment of the Bay of Bengal, India, showed maximum antifungal activity at pH 8.2 (42). Growth-limiting nutrients have a significant impact on the interaction between growth metabolism and product secretion in the formation of secondary metabolites (42). Carbohydrates such as starch, glycerol, maltose, sucrose, xylose, and mannose have been associated with the synthesis of maximal antimicrobial metabolites (43). Our recent work on *Streptomyces* sp. PBR11 also illustrates positive results for utilizing all the above-mentioned carbohydrates including trehalose, dextrose, l-arabinose, insulin, salicin, trehalose, mannitol, arbitol, erythritol, adonital, l-methyl-d-glucoside, rhamnose, cellubiose, melezitose, L-methyl-d-mannoside, xylitol, ONPG (*o*-nitrophenyl-β-d-galactopyranoside), esculin hydrolysis, d-arabinose, and sorbose. Eight different types of antibiotics from different classes such as quinolones, cephalosporins, beta-lactam first-generation cephalosporins, aminoglycosides, quinolones, nitrofurans, penicillin beta-lactam antibiotics, and sulfonamides were used to check the antibiotic resistant and susceptibility profiles of the strain using a disc diffusion method. The strain exhibited high sensitivity to norfloxacin (10 μg), cefotaxime (30 μg), cephalothin (30 μg), gentamicin (10 μg), and nalidixic acid (30 μg), whereas it showed resistance to nitrofurantoin (300 μg), ampicillin (10 μg), and co-trimoxazole (25 μg), respectively.

The class *Actinomycetia* continues to offer novel secondary metabolites with diverse biological activities, including antimicrobial, anticancer, and anthelmintic actions. These compounds have the potential to emerge as therapeutic guides for the synthesis of new chemical compounds. Several published reports have been documented that describe the GC-MS studies to identify the chemical constituents of bioactive compounds and natural product discovery (44, 45). Here, GC-MS analysis was performed in which five major compounds were detected in EtAc-PBR11 with various retention times. Phenolic compounds are potent antioxidants and exhibited significant antibacterial activity because of their lipophilic nature, which enhances their antimicrobial activity by promoting their contact with the cell membrane (46). GC-MS fractions with a more area percentage of phenolic compounds demonstrated strong antimicrobial activity (47). Phenol, 2,5-bis(1,1-dimethylethyl), is the major chemical compound detected in the EtAc-PBR11 with 33.03% (area %). This phenolic compound may significantly influence the inhibitory action against various test pathogens. Other major compounds include 1-tetradecanol, *n*-pentadecanol, 1-nonadecene, and pyrrolo[1,2-*a*]pyrazine-1,4-dione hexahydro-3-(phenylmethyl). These compounds have demonstrated significant antibacterial (48–50), antituberculosis, anticancer, antioxidant, antifungal, and antimicrobial activities, respectively (51–55).

The identification of the isolate PBR11 was done using the recommended international standards. The polyphasic approach has recently become the most widely accepted system for classification and identification (56–58). This method incorporates many different types of data, including phenotypic, chemotaxonomic, genotypic, and phylogenetic data (59). The phenotypic, microscopic, and biochemical properties of PBR11 were determined in accordance with the International Streptomyces Project (ISP) (60) and *Bergey’s Manual of Systematic Bacteriology* (61). The isolate was identified to the genus level by comparing the morphology of spore bearing hyphae with the entire spore chain and structure of spore as described by Li et al. (59). The opaque, rough, nonspreading morphology of *Streptomyces* colonies, which are frequently embedded and adhere to agar medium, make them easy to identify, and almost every colony of *Streptomyces* produced an earthy odor (geosmin), which is also an important characteristic of *Streptomyces* (62). Moreover, the phylogenetic analysis by a neighbor-joining approach showed that the PBR11 forms a separate subclade from the other members of *Streptomyces* sp., indicating that it may represent a novel taxon, and demonstrated 92.91% sequence similarity with the closest match, Streptomyces atrovirens NRRL B-16357. Therefore, the phenotypic, biochemical, and molecular traits of PBR11 revealed that the isolate possessed typical characteristics of the genus *Streptomyces*; thus, the isolate was designated *Streptomyces* sp. PBR11.These findings suggested that the PWS is a repository of novel *Streptomyces* sp. from the phylum *Actinomycetota* with promising antagonistic properties against various disease-causing pathogens.

Biosynthetic gene cluster PKS-II plays a central role in the biosynthesis of bioactive secondary metabolites (63). PBR11 strain exhibits positive results for the PKS type II gene. Aromatic polyketides, such as anthracycline, angucycline, and tetracycline, are commonly synthesized by polyketide synthase type II (64). The amino acid sequence of the PKS-II gene showed close similarity to beta-ketoacyl synthase (KS) family protein (99.42%) of *Streptomyces* sp. The predicted protein product showed maximum similarity to macrolide antibiotic megalomicin. The KS domain is the most conserved catalytic domain involved in type II polyketide synthesis. They are responsible for producing antimicrobial compounds with broad-spectrum antimicrobial activity (65).

Furthermore, the strain was also found positive for the chitinase GH18 family gene. Chitinases belong to the chitinase GH18 family gene, and they catalyze the hydrolysis of β-1,4-linkages in chitin which exhibited an inhibitory action on fungal growth (66). The amino acid sequence of the chitinase gene showed maximum similarity to the chitinase GH18 protein (98.18%) of Streptomyces katrae, and the predicted protein structures showed similarity to the crystal structure of chitinase 40 from thermophilic actinomycetia, Streptomyces thermoviolaceus, involved in the hydrolysis of the β-1,4-linkage between *N*-acetyl-d-glucosamine residues of chitin. The broad-spectrum antimicrobial activity of the *Streptomyces* sp. PBR11 strain may be controlled by the expression of the biosynthetic PKS type II gene and the chitinase GH18 family gene.

DMSO is an ideal solvent for preparing samples for evaluating biological activity and dissolving bioactive compounds in *in vitro* drug discovery (67, 68). Because of its ability to dissolve both polar and nonpolar compounds, as well as its miscibility with water and culture media, DMSO is an excellent solvent for bioassays (69). It also improves the accuracy of the concentrations of test compound or drug by reducing room temperature and evaporation and can be used to maintain stock solutions of test compounds (69, 70). For our studies, DMSO was used as a positive vehicle control and also for the preparation of crude extracts and bioactive fractions. Fractionation of the crude ethyl acetate extract EtAc-PBR11 produced two bioactive fractions, PBR11Fr-1 and PBR11Fr-2. Nonetheless, PBR11Fr-1 was the most promising fraction for antimicrobial activity. LC-MS/MS analysis of both bioactive fractions demonstrated the presence of antimicrobial and bioactive compounds. Three biologically active compounds—*N*-depyridomethyl-indinavir, crustecdysone (20-hydroxyecdysone), and ethambutol—were detected in both fractions. Indinavir, an antiretroviral drug, is a potent and specific inhibitor of HIV-1 (71, 72). According to a recent report (73), crustecdysone (20-hydroxyecdysone) demonstrated in many *in vitro* and *in vivo* models numerous biological properties, including anabolic, anti-inflammatory, antioxidant, immunomodulatory, antidiabetic, and anti-obesity activities, in addition to serving as a neuroprotective and hepatoprotective drug. It also has antiparasitic activity, inhibits caspase activity, and induces autophagy (74, 75). Ethambutol is a first-line drug therapy recommended by the World Health Organization for treating tuberculosis (76, 77). In the present investigation, ethambutol was detected for the first time in the bioactive fraction of *Streptomyces* sp. PBR11. This is the most significant finding that emerged from our study. Synthesizing this antituberculosis drug from any natural resources has not been reported so far. Other bioactive compounds detected in the fraction PBR11Fr-1 were arecoline, 10-nitro-9*Z*,12*Z*-octadecadienoic acid, campestanol, SM(d18:0/0:0), ethoxyquin, phendimetrazine, colforsin, 10-deoxymethymycin, anandamide (20:2, *n*-6), and miltefosine. Arecoline is reported to have anthelmintic activity (78, 79), while 10-nitro-9*Z*,12*Z*-octadecadienoic acid (80) and colforsin (81, 82) are known for displaying anti-inflammatory and apoptosis action. Campestanol (83), SM(d18:0/0:0) (84), ethoxyquin (85, 86), phendimetrazine (87), 10-deoxymethymycin (88), anandamide (20:2, *n*-6) (89), and miltefosine (90) have hypocholesterolemic, cell signaling, antioxidant, anticarcinogenic, anorexigenic, antibacterial, fatty acid neurotransmitter, and antileishmanial effects, while no bioactivity has been reported for 11-amino-undecanoic acid, d-pantetheine 4′-phosphate, and 3-hydroxytetradecanedioic acid. The biologically active compounds diethylcarbamazine, acetylsalicylic acid (aspirin), 2-propyl-9*Z*-octadecenoic acid, and oleandrin (72, 91–95) detected in the bioactive fraction of PBR11Fr-2 exhibited anti-inflammatory effects, nonsteroidal anti-inflammatory action, antiplasmodial activity, anticancer effects, novel antiviral activity, and anorexigenic effects. However, there is no bioactivity reported for 12,14-pentacosadiynoic. Our results confirmed that natural product research continues to be important in discovering and developing novel bioactive compounds for clinical and therapeutic applications.

The metabolites isolated from the *Streptomyces* sp. exhibited significant antimicrobial activity with low MIC values, making it one of the best sources of effective antimicrobial agents for treating infectious diseases (96). The MIC values exhibited by the crude extract EtAc-PBR11 and bioactive fraction PBR11Fr-1 were found within the range of ≥0.048 to ≥0.390 μg/mL. EtAc-PBR11 had the lowest MIC of ≥0.097 g/mL against C. albicans MTCC 227, while PBR11Fr-1 showed an MIC of ≥0.048 μg/mL. Similarly, against E. coli ATCC BAA-2469, the lowest MIC shown by EtAc-PBR11 was ≥0.390 μg/mL, and for PBR11Fr-1 it was ≥0.195 μg/mL, respectively. Against A. baumannii ATCC BAA-1705, the crude extract exhibited an MIC of ≥0.195 μg/mL, and the bioactive fraction showed an MIC of *≥*0.097 μg/mL. We found that the MICs displayed by the crude extract and the bioactive fraction PBR11Fr-1 were significantly lower than the MICs obtained from the standard antimicrobial drug. According to an earlier report (97), a compound isolated from the actinomycetia strain *M. auratinigra* showed promising antimicrobial activity with low MICs compared to the reference antibiotic neomycin sulfate.

SEM is a powerful tool for investigating changes in the bacterial surface structure and cell damage (98). In this study, the SEM result confirms the disruptive action of fraction PBR11Fr-1. It caused considerable morphological changes in the cellular membrane of the selected pathogens, including the shrinkage of the cells and surface deformation compared to untreated cells. This further indicates that the mode of bactericidal action of the bioactive fraction against the pathogens is through membrane disruption mechanism and prevents further cell development.

The drug toxicity of the bioactive fraction PBR11Fr-1 was evaluated against normal liver cell line CC1 using an MTT assay. The toxicity of the fraction was found to be dose dependent and demonstrated a low cytotoxic effect. The percentage of the cell viability started declining with the increase in the concentration from 50 μg/mL (87.07% ± 3.22%) to 100 μg/mL (81.26% ± 2.99%). Similar findings have been reported (39, 99), suggesting that *Streptomyces* spp. have fewer cytotoxic effects against normal cell lines.

## MATERIALS AND METHODS

### Soil sample collection site.

**FIG 8:**
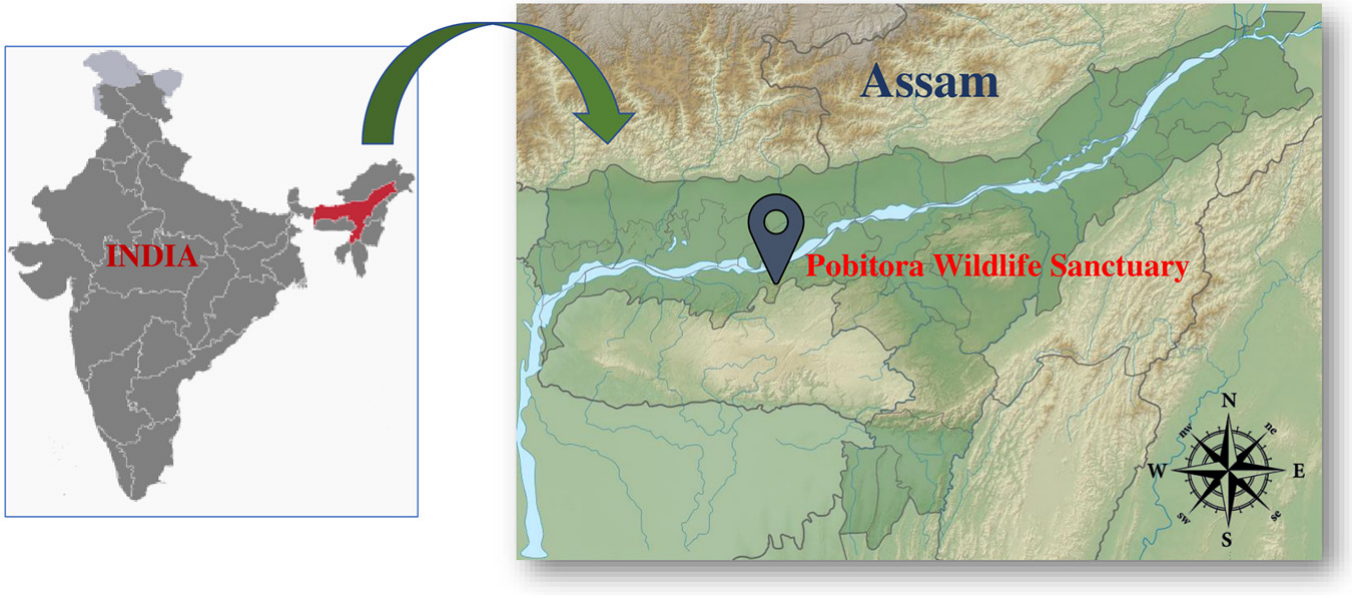
Soil sample collection site. (Maps from Wikimedia Commons.)

Samples of rhizosphere soil were collected from various sites of PWS (26°12″ to 26°16″N and 91°58″ to 92°05″E) of Assam, which is in the northeastern part of India (Fig. 8). The entire area falls under the Brahmaputra flood plains. Soil samples were taken from various depths on the earth’s surface, ranging from layers just beneath the upper surface to a 10-cm depth after removing the upper layer of the topsoil. The samples were collected in sterile zip lock bags, labeled correctly with the collection date, and moved to the laboratory on the same day. The collected soil samples were stored at 4°C until they were analyzed.

### Isolation of actinomycetes from soil samples.

The PBR11 strain was isolated from soil by the serial dilution technique (4). A soil suspension was made from the collected soil sample by dissolving 5 g of soil in 100 mL of normal saline water (NaCl 9g/l), which was then incubated at 28°C for 24 h at 180 rpm with continuous shaking. The mixtures were allowed to settle, and dilutions were made up to a concentration of 10-4 with sterile saline water and thoroughly mixed by vortexing at maximum speed. Aliquots (0.1 mL) from each dilution were spread onto plates of isolation media, followed by incubation at 28°C and checked after 48, 72, and 96 h (23). To inhibit the growth of fungi and bacteria, the isolation plates were supplemented with amphotericin B (75 μg/mL) and rifampicin (2.5 μg/mL), respectively. The culture plates were then incubated for 5 days at 28°C. Repeated streaking of the isolate on GLM (yeast extract, 3 g; malt extract, 3 g; peptone type I, 5 g; starch, 10 g; agar, 20 g; distilled water, 1,000 mL; pH 7.5) agar plates resulted in the purification of the PBR11 isolate, which was stored at –80°C in 20% glycerol for future use.

### Evaluation of antimicrobial activity. (i) Test pathogens.

The following test pathogens were used for the experiment. For Gram-positive bacteria, we used Staphylococcus aureus MTCC 96, MRSA ATCC 43300, Bacillus subtilis MTCC 441, and Micrococcus luteus MTCC 1538. For Gram-negative bacteria, we used Escherichia coli MTCC 739, Escherichia coli ATCC BAA-2469, Acinetobacter baumannii ATCC BAA-1705, Klebsiella pneumoniae MTCC 3384, and Pseudomonas aeruginosa MTCC 741. Two multidrug-resistant (MDR) clinical isolates—GNR18 (Pseudomonas aeruginosa) and GNR19 (Escherichia coli)—were obtained from Pathology Department, Guwahati Neurological Research Center, Guwahati, Assam, India, for the evaluation of the antimicrobial assay. We also used the yeast strains Candida albicans MTCC 227 and Candida tropicalis MTCC 184 and the fungus strains Aspergillus niger MTCC 282, Aspergillus fumigatus MTCC 1811, Trichophyton mentagrophytes MTCC 8476, T. rubrum MTCC 8477, T. tonsurans MTCC 8475, and Microsporum gypseum MTCC 8469. The MTCC strains were obtained from the Microbial Type Culture Collection, IMTECH, Chandigarh, India, and the ATCC strains were procured from HiMedia, Mumbai, India.

### (ii) Antimicrobial activity assessment.

Preliminary screening for *in vitro* antimicrobial bioassay of PBR11 strain against the test pathogens was carried out by the spot inoculation method (100). The zone of inhibition was evaluated after 24 to 48 h of incubation at 37°C for bacteria and at 28°C for yeasts and fungi. Each experiment had three replicates. The secondary screening for the antimicrobial assay was done by the disc diffusion method (101). The strain was grown in 250-mL flasks containing 100 mL of GLM broth and incubated for 5 days at 28°C and pH 7.5 in a rotary shaker (180 rpm) with continuous shaking. By centrifuging (Sigma 3K30) the culture broth at 7,000 × *g* for 20 min, the cell mass was separated. The supernatant was then extracted for 30 min in a separating funnel with ethyl acetate (EtAc) at a 1:1 (vol/vol) ratio. A rotary evaporator (Rotavapor R-215; BUCHI, Labortechnik AG, Flawil, Switzerland) was used to evaporate the EtAc extract to dryness under reduced pressure at 45°C. The dried extract (EtAc-PBR11) was prepared before the antimicrobial bioassay by dissolving it in 10% dimethyl sulfoxide (DMSO) at a concentration of 1 mg/mL. Then, 30 μL of the extract was loaded onto sterile discs (6 mm in diameter) placed on Mueller-Hinton agar plates seeded with bacterial pathogens (0.5 McFarland turbidity standards). For fungal pathogens, this was carried out on Sabouraud dextrose agar plates. Rifampicin (30 μg/disc) for bacteria and amphotericin B (30 μg/disc) for fungal species were used as positive controls, while a 10% DMSO-loaded disc served as a negative control. After 24 h of incubation at 37°C for bacteria and 28°C for *Candida*, antimicrobial activity was observed.

### (iii) Biochemical characterization and optimization of cultural conditions on growth and antimicrobial production.

The PBR11 strain was grown in nine different growth mediums to assess its cultural characteristics. Light microscopy and SEM were used to examine the pattern of the mycelium and ornamentation of the spore chain (16). The carbohydrate utilization by the strain was tested by using a Hicarbo kit (HiMedia). The PBR11 strain was grown in the Hicarbo kit and incubated at 28°C for 24 to 48 h. A disc diffusion method with Octa-Disc (HiMedia) was used to detect the susceptibility of the PBR11 strain to eight standard antibiotics. To evaluate the growth response and antibiotic synthesis, the strain was inoculated in GLM broth at 28°C for 8 days at various pH values ranging from acidic to alkaline (pH 3 to pH 12). The impact of temperature on the growth and production of the antimicrobial agent was investigated on GLM at different temperatures (15, 20, 25, 28, 32, 35, 40, and 45°C) at pH 8. The antimicrobial activity of PBR11 was assessed against C. albicans by the disc diffusion method (101) because C. albicans proved to be a more sensitive test organism during the evaluation of antimicrobial production by spot inoculation and well diffusion techniques.

### (iv) Gas chromatography-mass spectrometry analysis.

The crude extract (EtAc-PBR11) was dissolved in HPLC-grade methanol and filtered through a 0.2-μm syringe filter. The bioactive metabolites present in the EtAc-PBR11 were identified using GC-MS (16) with slight modifications. The peaks were identified by comparing the mass spectra to the NIST (USA) library.

### Molecular characterization. (i) 16S rRNA gene amplification and phylogenetic analysis.

A Genetix kit was used for genomic DNA extraction of the PBR11 strain. The universal eubacterial primers 27F (5′-AGA GTT TGA TCC TGG CTC AG-3′) and 1492R (5′-GGT TAC CTT GTT ACG ACT T-3′) were used to amplify the 16S rRNA gene (102). PCRs were carried out in a Proflex PCR System (Applied Biosystems, USA) by the method described by Sharma et al. (28). Then, a 1.8% (wt/vol) agarose gel prepared in 1× TAE buffer was used to confirm the amplified products. The PCR bands were analyzed using a Bio-Rad Gel Doc XR+ system (Hercules, Richmond, CA). BLASTN (103) and EzTaxon server (http://www.ezbiocloud.net) (104) were used for phylogenetic analysis based on the 16S rRNA gene sequence of the PBR11 strain. The top 40 nucleotide sequences of 16S rRNA genes with the highest nucleotide similarity were selected for multiple sequence alignment by the CLUSTAL W program (105). A phylogenetic tree was constructed by the neighbor-joining method (106) using MEGA X (107). A bootstrap analysis with 1,000 replications was used to assess the support of each clade (108).

### (ii) Detection and analysis of the PKS-II gene and chitinase gene for prediction of bioactive chemical classes.

To detect the antimicrobial metabolite-producing biosynthetic gene in *Streptomyces* sp. PBR11 strain, PCR amplification of polyketide synthase II (PKS-II) pathway-encoded genes was done. The PKS-II gene was amplified using the degenerate primers KS_α_F (5′-TSG CST GCT TCG AYG GCS ATC-3′) and KS_β_R (3′-TCG CCG BAA GCC GCC NAA GGT-5′) (109). Group A bacterial chitinase gene of glycoside hydrolase family 18 was amplified by using a degenerate set of primers, GA1F and GA1R, as previously described by (110). The PCRs for the PKS-II and chitinase genes were performed according to previously described methods (24, 111). The amplified products for the PKS-II and chitinase genes were evaluated in a 1.8% (wt/vol) agarose gel made in 1× TAE buffer. The PCR bands were examined under UV light and documented using a Bio-Rad Gel Doc XR+ system (Hercules). The PCR-amplified product of the PKS-II gene and the chitinase gene of the *Streptomyces* sp. PBR11 was sequenced at First BASE Laboratories, Malaysia. The web tool ORF FINDER was used to translate the nucleotide sequences of the PKS-II and chitinase genes into amino acid sequences, which were further used as queries to search their gene products in the NCBI database using the protein BLAST tool with default settings. The predicted pathway product of the biosynthetic gene of strain PBR11 was identified using SBSPKSv2 (112). The secondary structures of the polyketide ketosynthase domains and the chitinase gene were created using the PDBsum tool (113).

### (iii) Cultivation and fermentation of *Streptomyces* sp. PBR11 for the production of active metabolites.

The fermentation process of the strain *Streptomyces* sp. PBR11 was already discussed in the antimicrobial assay section. The fermentation was carried out for 3 days at pH 8 under identical conditions (28). A total of 20 L of culture broth was prepared by continuing the fermentations, in the same way, to extract and purify the active metabolites. The harvested cell mass of *Streptomyces* sp. PBR11 was separated by centrifugation at 7,000 × *g* for 15 min, and the supernatant was extracted twice with equal volumes of EtAc-fermented broth (1:1 [vol/vol]) for 30 min in a separating funnel. A rotary evaporator (Rotavapor-R 210; BUCHI) was used to dry the EtAc extract of *Streptomyces* sp. PBR11 (EtAc-PBR11) under reduced pressure at 45°C. The dried crude extract was collected and stored in a refrigerator for further investigation.

### (iv) Bioactivity-guided fractionation and chemical profiling of *Streptomyces* sp. PBR11.

Three chromatographic methods—TLC, flash chromatography, and LC-MS/MS—were used to investigate the metabolomic profile of EtAc-PBR11.

### (v) Thin-layer chromatography.

Silica gel-coated TLC plates (TLC silica gel 60 F254; Merck, Germany) were used to evaluate the polarity of the secondary metabolites present in EtAc-PBR11. The nature of the secondary metabolites and the pattern of separation of the various fractions formed by them were evaluated by dissolving the dried crude extract in methanol and then examining it on TLC plates. The extract was overlaid onto a TLC plate by repeated spotting with a small capillary at room temperature in a TLC chamber. The loaded plate was run in various combinations of organic solvent systems—*viz.* chloroform-methanol, ethyl acetate-methanol, and hexane-ethyl acetate—and visualized under visible and UV light. After development, the TLC plate was stained to identify the various colored bands of the compounds in the crude extracts using anisaldehyde/H_2_SO_4_ spraying reagent.

### (vi) Flash chromatography analysis.

The bioactive crude extract from the culture broth of *Streptomyces* sp. strain PBR11 was fractionated by using an automated flash chromatography system (RF+LUMEN UV-VIS; CombiFlash, USA) on a silica (RediSep, Rf 40 to 60 μm) column through step gradient elution by using hexane, chloroform, and methanol, and a total of 195 fractions (15 mL each) were collected. Based on their separation pattern, similar fractions were combined as follows: CFr.1 (fractions 1 to 20), CFr.2 (fractions 21 to 47), CFr.3 (fractions 48 to 77), CFr.4 (fractions 78 to 105), CFr.5 (fractions 106 to 149), and CFr.6 (fractions 150 to 195). Based, on the bioactivity analysis, it was found that the CFr.5 (fractions 106 to 149) was the most bioactive fraction, while the rest of the fractions were either poorly active or inactive. Hence, bioactive fraction CFr.5 was subjected to fractionation by using chloroform and methanol and a total of 72 fractions (15 mL each) were collected. The chromatographic separation pattern of the various fractions was checked by TLC using various solvent systems and, based on their separation patterns, similar fractions were combined in the following manner: CCFr.1 (CFr.1-10), CCFr.2 (CFr.11-28), CCFr.3 (CFr.29-46), and CCFr.4 (CFr.47-72). Analysis of the bioactivity of each of the combined fractions was done, and CCFr.2 (CFr.11-28) had the highest bioactivity, while the rest of them were either poorly active or inactive. Based on the TLC analysis of the CCFr.2 (CFr.11-28) fraction, it was observed that it has two major spots, and therefore was subjected to preparative TLC, and two fractions were collected (named PBR11Fr-1 and PBR11Fr-2). Both fractions were analyzed for their bioactivity.

### (vii) LC-MS/MS analysis.

The chemical compounds present in the bioactive fractions of PBR11Fr-1 and PBR11Fr-2 were identified using LC-MS/quadrupole time-of-flight mass spectrometry (410 Prostar Binary LC with 500 MS; Varian, Inc., USA) equipped with a photodiode array detector and a C_18_ column. Both the fractions were dissolved in HPLC-grade methanol, and the injection volume was 3 μL. The mobile phases employed for the analysis were A (100% water) and B (100% acetonitrile) in a gradient elution method with a 30-min run time and a flow rate of 0.3 mL/min. Data on mass spectra were retrieved in positive ionization mode for the mass range *m/z* 150 to 1,000. In addition, the following conditions were set for the mass spectrometer: nebulizing gas pressure, 35 lb/in^2^; drying gas flow rate, 13 L/min; drying gas temperature, 300°C; and nebulizing gas flow rate, 11 L/min.

### (viii) Investigation of MIC of EtAc-PBR11 and PBR11Fr-1.

The MIC assay was carried out according to the previous method, with a few modifications (114). A stock solution (1,000 μg/mL) of filter-sterilized (0.2 μm) EtAc-PBR11 and bioactive fraction PBR11Fr-1 was prepared in 10% DMSO, and 2-fold serial dilutions of the extracts (working solution, 50 to 0.048 μg/mL) were prepared for MIC tests. These were transferred to a 96-well plate, and each well received 10^5^ CFU (0.5 McFarland turbidity standards) of freshly grown test microbial strains. The positive control, made with conventional antibiotics such as levofloxacin and amphotericin B, should be clear, while the negative control made with DMSO (10%) without antimicrobial drugs, should be turbid. The bacteria and *Candida* plates were incubated aseptically for 24 h at 37 and 28°C, respectively, and the absorbance at 600 nm was measured by using a UV-Vis spectrophotometer. (Varioskan Flash; Thermo Scientific, San Jose, CA). After adding 30 μL of 0.015% resazurin to each well, the plates were incubated for 2 h at room temperature. The MIC value of the extract was determined by the concentration at which blue was observed. Cells (10 μL) from the blue-colored wells were spread on Muller-Hinton agar and Sabouraud dextrose agar plates, followed by incubation overnight. MICs were defined as the values at which no visible microbial growth was seen.

### (ix) Morphological effects of PBR11Fr-1 on test pathogens.

The bioactive fraction PBR11Fr-1 was studied by SEM as described earlier (28) with a few modifications for its antimicrobial effects on C. albicans MTCC 227, E. coli ATCC BAA-2469 and A. baumannii ATCC BAA-1705. Test microorganisms were treated with 1× MIC PBR11Fr-1.

### (x) *In vitro* cytotoxic activity of bioactive fraction PBR11Fr-1.

The *in vitro* drug sensitivity of the bioactive fraction, PBR11Fr-1, was assessed by 3-(4,5-dimethylthiazol-2-yl)-2,5-diphenyl tetrazolium bromide (MTT) assay. In 48-well plates, normal liver cells (CC-1) were seeded at a density of 2,000 cells per well. These were allowed to grow overnight and then treated with PBR11Fr-1 at various concentrations (5, 10, 25, 50, 75, and 100 μg/mL) for 48 h at 37°C in a humidified 5% CO_2_ incubator. An MTT reduction conversion assay was used to assess drug cytotoxicity. Each well received 50 μL of MTT at a concentration of 5 mg/mL and was incubated for 4 h. Formazan crystals formed by mitochondrial enzymatic action on the MTT substrate were solubilized in 200 μL of DMSO. After the plates were shaken for 20 min, the absorbance at 570 nm was measured by using a microplate reader. Each treatment was performed in triplicate. All data are presented as means ± the standard deviations (SD). The number of healthy cells in a sample is known as the cell viability. The evaluation of cell viability is essential in toxicity studies. Cell viability assays are primarily used to test the response of cells to a drug or chemical agent (115, 116). The following equation was used to calculate the percentage of cell viability:

$$\text{\% cell viability }=\;\left(\frac{A_{\text{treatment}}-\;A_{\text{blank}}}{A_{\text{control}}-\;A_{\text{blank}}}\right)\;\times \text{ 1}00,$$

where, *A* = absorbance.

### Bioinformatics and data analysis.

All experiments were performed in biological triplicate. The data are reported as means ± the standard deviations of the mean. The chemical structure and other parameters for each compound were searched using online database software. MarvinSketch was used to draw the chemical structures.

### Data availability.

The partial 16S rRNA gene sequence of the PBR11 strain was deposited into the NCBI GenBank database under the accession number MH718314. The strain showed the highest sequence similarity with Streptomyces atrovirens strain NRRL B-16357 (DQ026672). The partial sequences of the PKS-II gene and the chitinase gene were deposited in GenBank under accession numbers ON911582 and ON911583, respectively.

## References

[1] Qadri H, Shah AH, Mir M. 2021. Novel strategies to combat the emerging drug resistance in human pathogenic microbes. Curr Drug Targets 22:1424–1436. doi:10.2174/1389450121666201228123212. 33371847

[2] Jackson N, Czaplewski L, Piddock LJ. 2018. Discovery and development of new antibacterial drugs: learning from experience? J Antimicrob Chemother 73:1452–1459. doi:10.1093/jac/dky019. 29438542

[3] Salam N, Jiao JY, Zhang XT, Li WJ. 2020. Update on the classification of higher ranks in the phylum *Actinobacteria*. Int J Syst Evol Microbiol 70:1331–1355. doi:10.1099/ijsem.0.003920. 31808738

[4] Sharma P, Thakur D. 2020. Antimicrobial biosynthetic potential and diversity of culturable soil actinobacteria from forest ecosystems of Northeast India. Sci Rep 10:1–8. doi:10.1038/s41598-020-60968-6. 32139731 PMC7057963

[5] Cross T. 1981. Aquatic actinomycetes: a critical survey of the occurrence, growth and role of actinomycetes in aquatic habitats. J Appl Bacteriol 50:397–423. doi:10.1111/j.1365-2672.1981.tb04245.x. 7019182

[6] Bull AT, Ward AC, Goodfellow M. 2000. Search and discovery strategies for biotechnology: the paradigm shift. Microbiol Mol Biol Rev 64:573–606. doi:10.1128/MMBR.64.3.573-606.2000. 10974127 PMC99005

[7] Goodfellow M, Nouioui I, Sanderson R, Xie F, Bull AT. 2018. Rare taxa and dark microbial matter: novel bioactive actinobacteria abound in Atacama Desert soils. Antonie Van Leeuwenhoek 111:1315–1332. doi:10.1007/s10482-018-1088-7. 29721711

[8] Barka EA, Vatsa P, Sanchez L, Gaveau-Vaillant N, Jacquard C, Meier-Kolthoff JP, Klenk H-P, Clément C, Ouhdouch Y, van Wezel GP. 2016. Taxonomy, physiology, and natural products of Actinobacteria. Microbiol Mol Biol Rev 80:1–43. doi:10.1128/MMBR.00019-15. 26609051 PMC4711186

[9] Rahlwes KC, Sparks IL, Morita YS. 2019. Cell walls and membranes of *Actinobacteria*. Subcell Biochem 93:417–469. doi:10.1007/978-3-030-18768-2_13. 31214994

[10] Risdian C, Mozef T, Wink J. 2019. Biosynthesis of polyketides in *Streptomyces*. Microorganisms 7:124. doi:10.3390/microorganisms7050124. 31064143 PMC6560455

[11] Miao V, Davies J. 2010. Actinobacteria: the good, the bad, and the ugly. Antonie Van Leeuwenhoek 98:143–150. doi:10.1007/s10482-010-9440-6. 20390355

[12] Sohda KY, Nagai K, Yamori T, Suzuki K, Tanaka A. 2005. YM216391, a novel cytotoxic cyclic peptide from *Streptomyces nobilis*. I. Fermentation, isolation, and biological activities. J Antibiot (Tokyo) 58:27–31. doi:10.1038/ja.2005.2. 15813177

[13] Nguyen HT, Pokhrel AR, Nguyen CT, Pham VT, Dhakal D, Lim HN, Jung HJ, Kim TS, Yamaguchi T, Sohng JK. 2020. *Streptomyces* sp. VN1, a producer of diverse metabolites including non-natural furan-type anticancer compound. Sci Rep 10:1–4. doi:10.1038/s41598-020-58623-1. 32019976 PMC7000394

[14] Harvey AL, Edrada-Ebel R, Quinn RJ. 2015. The re-emergence of natural products for drug discovery in the genomics era. Nat Rev Drug Discov 14:111–129. doi:10.1038/nrd4510. 25614221

[15] Hayashi Y, Yamazaki-Nakamura Y, Yakushiji F. 2013. Medicinal chemistry and chemical biology of diketopiperazine-type antimicrotubule and vascular-disrupting agents. Chem Pharm Bull (Tokyo) 61:889–901. doi:10.1248/cpb.c13-00404. 23995353

[16] Russo P, Del Bufalo A, Fini M. 2015. Deep sea as a source of novel-anticancer drugs: update on discovery and preclinical/clinical evaluation in a systems medicine perspective. Excli J 14:228–236. doi:10.17179/excli2014-632. 26600744 PMC4652633

[17] Romero F, Espliego F, Pérez Baz JP, García de Quesada T, Grávalos D, De La Calle FE, Fernández-Puentes JL. 1997. Thiocoraline, a new depsipeptide with antitumor activity produced by a marine *Micromonospora* I. Taxonomy, fermentation, isolation, and biological activities. J Antibiot (Tokyo) 50:734–737. doi:10.7164/antibiotics.50.734. 9360617

[18] Renner MK, Shen YC, Cheng XC, Jensen PR, Frankmoelle W, Kauffman CA, Fenical W, Lobkovsky E, Clardy J. 1999. Cyclomarins A to C, new anti-inflammatory cyclic peptides produced by a marine bacterium (*Streptomyces* sp.). J Am Chem Soc 121:11273–11276. doi:10.1021/ja992482o.

[19] Walsh CT. 2003. Antibiotics: actions, origin, resistance. ASM Press, Washington, DC. doi:10.1110/ps.041032204.

[20] Onaka H. 2017. Novel antibiotic screening methods to awaken silent or cryptic secondary metabolic pathways in actinomycetes. J Antibiot 70:865–870. doi:10.1038/ja.2017.51. 28442735

[21] Okazaki T, Naito A. 1986. Studies on actinomycetes isolating from Australian soil. Biol Biochem Biomed Aspects Actinomycetes 1986:739–741.

[22] Saadoun I, Gharaibeh R. 2003. The *Streptomyces* flora of Badia region of Jordan and its potential as a source of antibiotic-resistant bacteria. J Arid Environ 53:365–371. doi:10.1006/jare.2002.1043.

[23] Thakur D, Yadav A, Gogoi BK, Bora TC. 2007. Isolation and screening of *Streptomyces* in soil of protected forest areas from the states of Assam and Tripura, India, for antimicrobial metabolites. J Med Mycol 17:242–249. doi:10.1016/j.mycmed.2007.08.001.

[24] Das R, Romi W, Das R, Sharma HK, Thakur D. 2018. Antimicrobial potentiality of actinobacteria isolated from two microbiologically unexplored forest ecosystems of Northeast India. BMC Microbiol 18:1–6. doi:10.1186/s12866-018-1215-7. 29996765 PMC6042205

[25] Harvey A. 2000. Strategies for discovering drugs from previously unexplored natural products. Drug Discov Today 5:294–300. doi:10.1016/S1359-6446(00)01511-7. 10856912

[26] Sayed AM, Hassan MH, Alhadrami HA, Hassan HM, Goodfellow M, Rateb ME. 2020. Extreme environments: microbiology leading to specialized metabolites. J Appl Microbiol 128:630–657. doi:10.1111/jam.14386. 31310419

[27] Berdy J. 2005. Bioactive microbial metabolites: a personal view. J Antibiot 58:1–26. doi:10.1038/ja.2005.1. 15813176

[28] Sharma P, Kalita MC, Thakur D. 2016. Broad spectrum antimicrobial activity of forest-derived soil actinomycete, *Nocardia* sp. PB-52. Front Microbiol 7:347. doi:10.3389/fmicb.2016.00347. 27047463 PMC4796592

[29] Ramesh B, Kumar SP. 2011. Determining population size and demography of Great Indian one-horned rhino-*Rhinoceros unicornis* in Pobitora Wildlife Sanctuary, Assam, India. NeBIO 2:14–19.

[30] Bruns A, Cypionka H, Overmann J. 2002. Cyclic AMP and acyl homoserine lactones increase the cultivation efficiency of heterotrophic bacteria from the central Baltic Sea. Appl Environ Microbiol 68:3978–3987. 12147499 10.1128/AEM.68.8.3978-3987.2002 PMC124024

[31] Chaudhary HS, Yadav J, Shrivastava AR, Singh S, Singh AK, Gopalan N. 2013. Antibacterial activity of actinomycetes isolated from different soil samples of Sheopur (a city of central India). J Adv Pharm Technol Res 4:118–123. doi:10.4103/2231-4040.111528. 23833752 PMC3696223

[32] Button DK, Schut F, Quang P, Martin R, Robertson BR. 1993. Viability and isolation of marine bacteria by dilution culture: theory, procedures, and initial results. Appl Environ Microbiol 59:881–891. 16348896 10.1128/aem.59.3.881-891.1993 PMC202203

[33] Chin KJ, Hahn D, Hengstmann UL, Liesack W, Janssen PH. 1999. Characterization and identification of numerically abundant culturable bacteria from the anoxic bulk soil of rice paddy microcosms. Appl Environ Microbiol 65:5042–5049. doi:10.1128/AEM.65.11.5042-5049.1999. 10543821 PMC91679

[34] Khaled Z. 2021. Characterization, identification, and optimization of chitinolytic rare actinomycetes isolated from Sinai soil, Egypt. AJPS 64:183–202. https://ajps.journals.ekb.eg/article_187825.html.

[35] Schoenborn L, Yates PS, Grinton BE, Hugenholtz P, Janssen PH. 2004. Liquid serial dilution is inferior to solid media for isolation of cultures representative of the phylum-level diversity of soil bacteria. Appl Environ Microbiol 70:4363–4366. 15240320 10.1128/AEM.70.7.4363-4366.2004 PMC444819

[36] Berdy J. 1989. The discovery of new bioactive microbial metabolites: screening and identification. Prog Ind Microbiol 27:3–27.

[37] Macura AB. 1993. *In vitro* susceptibility of dermatophytes to antifungal drugs: a comparison of two methods. Int J Dermatol 32:533–536. doi:10.1111/j.1365-4362.1993.tb02844.x. 8340195

[38] Gu D, Hatch M, Ghannoum M, Elewski BE. 2020. Treatment-resistant dermatophytosis: a representative case highlighting an emerging public health threat. JAAD Case Rep 6:1153–1155. doi:10.1016/j.jdcr.2020.05.025. 33134459 PMC7591325

[39] Sudha S, Masilamani SM. 2012. Characterization of cytotoxic compound from marine sediment derived actinomycete *Streptomyces avidinii* strain SU4. Asian Pac J Trop Biomed 2:770–773. doi:10.1016/S2221-1691(12)60227-5. 23569845 PMC3609218

[40] Jose PA, Maharshi A, Jha B. 2021. Actinobacteria in natural products research: progress and prospects. Microbiol Res 246:126708. doi:10.1016/j.micres.2021.126708. 33529791

[41] Narayana KJ, Vijayalakshmi M. 2008. Optimization of antimicrobial metabolites production by *Streptomyces albidoflavus*. Res J Pharmacol 2:4–7.

[42] Kumar S, Kannabiran K. 2010. Antifungal activity of *Streptomyces* VITSVK5 spp. against drug resistant *Aspergillus* clinical isolates from pulmonary tuberculosis patients. J Mycol Med 20:101–107. doi:10.1016/j.mycmed.2010.04.005.

[43] Demain AL, Fang A. 1995. Emerging concepts of secondary metabolism in actinomycetes. Actinomycetologica 9:98–117. doi:10.3209/saj.9_98.

[44] Ser HL, Ab Mutalib NS, Yin WF, Chan KG, Goh BH, Lee LH. 2015. Evaluation of antioxidative and cytotoxic activities of *Streptomyces pluripotens* MUSC 137 isolated from mangrove soil in Malaysia. Front Microbiol 6:1398. doi:10.3389/fmicb.2015.01398. 26733951 PMC4679926

[45] Tan LT, Ser HL, Yin WF, Chan KG, Lee LH, Goh BH. 2015. Investigation of antioxidative and anticancer potentials of *Streptomyces* sp. MUM256 isolated from Malaysia mangrove soil. Front Microbiol 6:1316. doi:10.3389/fmicb.2015.01316. 26635777 PMC4659911

[46] Sikkema JA, de Bont JA, Poolman B. 1995. Mechanisms of membrane toxicity of hydrocarbons. Microbiol Rev 59:201–222. doi:10.1128/mr.59.2.201-222.1995. 7603409 PMC239360

[47] Kumar PS, Duraipandiyan V, Ignacimuthu S. 2014. Isolation, screening and partial purification of antimicrobial antibiotics from soil *Streptomyces* sp. SCA 7. Kaohsiung J Med Sci 30:435–446. doi:10.1016/j.kjms.2014.05.006. 25224766 PMC11916805

[48] Hasturk H, Jones VL, Andry C, Kantarci A. 2007. 1-Tetradecanol complex reduces progression of *Porphyromonas gingivalis*-induced experimental periodontitis in rabbits. J Periodontol 78:924–932. doi:10.1902/jop.2007.060293. 17470028

[49] Yan PS, Song Y, Sakuno E, Nakajima H, Nakagawa H, Yabe K. 2004. Cyclo (l-leucyl-l-prolyl) produced by *Achromobacter xylosoxidans* inhibits aflatoxin production by *Aspergillus parasiticus*. Appl Environ Microbiol 70:7466–7473. doi:10.1128/AEM.70.12.7466-7473.2004. 15574949 PMC535151

[50] Chatterjee S, Karmakar A, Azmi SA, Barik A. 2018. Antibacterial activity of long-chain primary alcohols from *Solena amplexicaulis* leaves. *In* Proceedings of the Zoological Society, vol 71, no. 4, p 313–319. Springer India. doi:10.1007/s12595-017-0208-0.

[51] Rukachaisirikul T, Siriwattanakit P, Sukcharoenphol K, Wongvein C, Ruttanaweang P, Wongwattanavuch P, Suksamrarn A. 2004. Chemical constituents and bioactivity of *Piper sarmentosum*. J Ethnopharmacol 93:173–176. doi:10.1016/j.jep.2004.01.022. 15234750

[52] Lee YS, Kang MH, Cho SY, Jeong CS. 2007. Effects of constituents of *Amomum xanthioides* on gastritis in rats and on growth of gastric cancer cells. Arch Pharm Res 30:436–443. doi:10.1007/BF02980217. 17489359

[53] Smaoui S, Mathieu F, Elleuch L, Coppel Y, Merlina G, Karray-Rebai I, Mellouli L. 2012. Taxonomy, purification and chemical characterization of four bioactive compounds from new *Streptomyces* sp. TN256 strain. World J Microbiol Biotechnol 28:793–804. doi:10.1007/s11274-011-0872-6. 22805798

[54] Wang G, Dai S, Chen M, Wu H, Xie L, Luo X, Li X. 2010. Two diketopiperazine cyclo (pro-phe) isomers from marine bacteria *Bacillus subtilis* sp. 13-2. Chem Nat Compd 46:583–585. doi:10.1007/s10600-010-9680-8.

[55] Kumar SN, Mohandas C, Siji JV, Rajasekharan KN, Nambisan B. 2012. Identification of antimicrobial compound, diketopiperazines, from a *Bacillus* sp. N strain associated with a rhabditid entomopathogenic nematode against major plant pathogenic fungi. J Appl Microbiol 113:914–924. doi:10.1111/j.1365-2672.2012.05385.x. 22747978

[56] Vandamme P, Pot B, Gillis M, de Vos P, Kersters K, Swings J. 1996. Polyphasic taxonomy, a consensus approach to bacterial systematics. Microbiol Rev 60:407–438. doi:10.1128/mr.60.2.407-438.1996. 8801440 PMC239450

[57] Prakash O, Verma M, Sharma P, Kumar M, Kumari K, Singh A, Kumari H, Jit S, Gupta SK, Khanna M, Lal R. 2007. Polyphasic approach of bacterial classification: an overview of recent advances. Indian J Microbiol 47:98–108. doi:10.1007/s12088-007-0022-x. 23100651 PMC3450112

[58] Gannibal PB. 2022. Polyphasic approach to fungal taxonomy. Biol Bull Rev 12:18–28. doi:10.1134/S2079086422010029.

[59] Li Q, Chen X, Jiang Y, Jiang C. 2016. Morphological identification of actinobacteria, p 59–86. *In* Actinobacteria: basics and biotechnological applications doi:10.5772/61461.

[60] Shirling ET, Gottlieb D. 1966. Methods for characterization of *Streptomyces* species. Int J Syst Bacteriol 16:313–340. doi:10.1099/00207713-16-3-313.

[61] Locci R. 1989. *Streptomyces* and related genera, p 2541–2508. *In* Bergey’s manual of systematic bacteriology. Williams & Wilkins, Baltimore, MD.

[62] Intra B, Mungsuntisuk I, Nihira T, Igarashi Y, Panbangred W. 2011. Identification of actinomycetes from plant rhizospheric soils with inhibitory activity against *Colletotrichum* spp., the causative agent of anthracnose disease. BMC Res Notes 4:98–99. doi:10.1186/1756-0500-4-98. 21457542 PMC3080312

[63] Rutledge PJ, Challis GL. 2015. Discovery of microbial natural products by activation of silent biosynthetic gene clusters. Nat Rev Microbiol 13:509–523. doi:10.1038/nrmicro3496. 26119570

[64] Zhang Z, Pan HX, Tang GL. 2017. New insights into bacterial type II polyketide biosynthesis. F1000Res 6:172. doi:10.12688/f1000research.10466.1. 28299197 PMC5321127

[65] Selvin J, Sathiyanarayanan G, Lipton AN, Al-Dhabi NA, Valan Arasu M, Kiran GS. 2016. Ketide synthase (KS) domain prediction and analysis of iterative type II PKS gene in marine sponge-associated actinobacteria producing biosurfactants and antimicrobial agents. Front Microbiol 7:63. doi:10.3389/fmicb.2016.00063. 26903957 PMC4751271

[66] Oyeleye A, Normi YM. 2018. Chitinase: diversity, limitations, and trends in engineering for suitable applications. Biosci Rep 38. doi:10.1042/BSR20180323. PMC6131217 30042170

[67] Kreiling R, Gehrke H, Broschard TH, Dreessen B, Eigler D, Hart D, Höpflinger V, Kleber M, Kupny J, Li Q, Ungeheuer P, Sauer UG. 2017. In chemico, *in vitro* and *in vivo* comparison of the skin sensitizing potential of eight unsaturated and one saturated lipid compounds. Regul Toxicol Pharmacol 90:262–276. doi:10.1016/j.yrtph.2017.09.023. 28958912

[68] Ilouga PE, Winkler D, Kirchhoff C, Schierholz B, Wölcke J. 2007. Investigation of 3 industry-wide applied storage conditions for compound libraries. J Biomol Screen 12:21–32. doi:10.1177/1087057106295507. 17099243

[69] Balakin KV, Savchuk NP, Tetko IV. 2006. In silico approaches to prediction of aqueous and DMSO solubility of drug-like compounds: trends, problems and solutions. Curr Med Chem 13:223–241. doi:10.2174/092986706775197917. 16472214

[70] Cushnie TP, Cushnie B, Echeverría J, Fowsantear W, Thammawat S, Dodgson JL, Law S, Clow SM. 2020. Bioprospecting for antibacterial drugs: a multidisciplinary perspective on natural product source material, bioassay selection and avoidable pitfalls. Pharm Res 37:1–24. doi:10.1007/s11095-020-02849-1. 32529587

[71] Balani SK, Woolf EJ, Hoagland VL, Sturgill MG, Deutsch PJ, Yeh KC, Lin JH. 1996. Disposition of indinavir, a potent HIV-1 protease inhibitor, after an oral dose in humans. Drug Metab Dispos 24:1389–1394. 8971147

[72] Hoetelmans RM, Meenhorst PL, Mulder JW, Burger DM, Koks CH, Beijnen JH. 1997. Clinical pharmacology of HIV protease inhibitors: focus on saquinavir, indinavir, and ritonavir. Pharm World Sci 19:159–175. doi:10.1023/A:1008629608556. 9297727

[73] Osipova S. 2021. Anti-protozoal effect of steroid hormone 20-hydroxyecdysone. ClinicalTrials.gov. https://clinicaltrials.gov/ct2/show/NCT04827537.

[74] Mhashilkar AS, Adapa SR, Jiang RH, Williams SA, Zaky W, Slatko BE, Luck AN, Moorhead AR, Unnasch TR. 2016. Phenotypic and molecular analysis of the effect of 20-hydroxyecdysone on the human filarial parasite *Brugia malayi*. Int J Parasitol 46:333–341. doi:10.1016/j.ijpara.2016.01.005. 26896576 PMC4936774

[75] Liu H, Wang J, Li S. 2014. E93 predominantly transduces 20-hydroxyecdysone signaling to induce autophagy and caspase activity in *Drosophila* fat body. Insect Biochem Mol Biol 45:30–39. doi:10.1016/j.ibmb.2013.11.005. 24316411

[76] Yendapally R, Lee RE. 2008. Design, synthesis, and evaluation of novel ethambutol analogues. Bioorg Med Chem Lett 18:1607–1611. doi:10.1016/j.bmcl.2008.01.065. 18242089 PMC2276401

[77] Lee N, Nguyen H. 2021. Ethambutol. *In* StatPearls [Internet] StatPearls Publishing. Available from https://www.ncbi.nlm.nih.gov/books/NBK559050/.

[78] Yusuf H, Yong SL. 2002. Oral submucous fibrosis in a 12-year-old Bangladeshi boy: a case report and review of literature. Int J Paediatr Dent 12:271–276. doi:10.1046/j.1365-263x.2002.00373.x. 12121538

[79] Wang SW, Hwang GS, Chen TJ, Wang PS. 2008. Effects of arecoline on testosterone release in rats. Am J Physiol Endocrinol Metab 295:E497–E504. doi:10.1152/ajpendo.00045.2008. 18559981

[80] Wright MM, Schopfer FJ, Baker PR, Vidyasagar V, Powell P, Chumley P, Iles KE, Freeman BA, Agarwal A. 2006. Fatty acid transduction of nitric oxide signaling: nitrolinoleic acid potently activates endothelial heme oxygenase 1 expression. Proc Natl Acad Sci USA 103:4299–4304. doi:10.1073/pnas.0506541103. 16537525 PMC1449687

[81] Hayashida N, Chihara S, Tayama E, Takaseya T, Enomoto N, Kawara T, Aoyagi S. 2001. Anti-inflammatory effects of colforsin daropate hydrochloride, a novel water-soluble forskolin derivative. Ann Thorac Surg 71:1931–1938. doi:10.1016/S0003-4975(01)02531-0. 11426771

[82] Oishi H, Takano KI, Tomita K, Takebe M, Yokoo H, Yamazaki M, Hattori Y. 2012. Olprinone and colforsin daropate alleviate septic lung inflammation and apoptosis through CREB-independent activation of the Akt pathway. Am J Physiol Lung Cell Mol Physiol 303:L130–40. doi:10.1152/ajplung.00363.2011. 22610350

[83] Connor WE. 1968. Dietary sterols: their relationship to atherosclerosis. J Am Diet Assoc 52:202–208. doi:10.1016/S0002-8223(21)12113-3. 4966658

[84] Hannun YA. 1994. The sphingomyelin cycle and the second messenger function of ceramide. J Biol Chem 269:3125–3128. doi:10.1016/S0021-9258(17)41834-5. 8106344

[85] Lundebye AK, Hove H, Måge A, Bohne VJ, Hamre K. 2010. Levels of synthetic antioxidants (ethoxyquin, butylated hydroxytoluene and butylated hydroxyanisole) in fish feed and commercially farmed fish. Food Addit Contam Part A Chem Anal Control Expo Risk Assess 27:1652–1657. doi:10.1080/19440049.2010.508195. 20931417

[86] Wattenberg LW. 1972. Inhibition of carcinogenic and toxic effects of polycyclic hydrocarbons by phenolic anti-oxidants and ethoxyquin. J Natl Cancer Inst 48:1425–1430. doi:10.1093/jnci/48.5.1425. 5030956

[87] Rothman RB, Katsnelson M, Vu N, Partilla JS, Dersch CM, Blough BE, Baumann MH. 2002. Interaction of the anorectic medication, phendimetrazine, and its metabolites with monoamine transporters in rat brain. Eur J Pharmacol 447:51–57. doi:10.1016/S0014-2999(02)01830-7. 12106802

[88] Kinumaki A, Suzuki M. 1972. The structure of a new macrolide antibiotic, YC-17. J Chem Soc 1972:744–745. doi:10.1039/c39720000744.

[89] Dsouza J, Chakraborty A, Veigas J. 2020. Biological connection to the feeling of happiness. JCDR 14. doi:10.7860/JCDR/2020/45423.14092.

[90] Sundar S, Jha TK, Thakur CP, Engel J, Sindermann H, Fischer C, Junge K, Bryceson A, Berman J. 2002. Oral miltefosine for Indian visceral *leishmaniasis*. N Engl J Med 347:1739–1746. doi:10.1056/NEJMoa021556. 12456849

[91] Peixoto CA, Silva BS. 2014. Anti-inflammatory effects of diethylcarbamazine: a review. Eur J Pharmacol 734:35–41. doi:10.1016/j.ejphar.2014.03.046. 24726556

[92] Liu N, Mathews A, Swanson J, Mhaskar R, Mathews A, Ayoubi N, Mirza AS. 2020. Aspirin use for cardiovascular disease prevention in the uninsured population. SAGE Open Med 8:2050312120938224. doi:10.1177/2050312120938224. 32647578 PMC7328214

[93] Panda S, Rout JR, Pati P, Ranjit M, Sahoo SL. 2018. Antimalarial activity of *Artemisia nilagirica* against *Plasmodium falciparum*. J Parasit Dis 42:22–27. doi:10.1007/s12639-017-0956-9. 29491554 PMC5825358

[94] Wittayanarakul K, Hannongbua S, Feig M. 2008. Accurate prediction of protonation state as a prerequisite for reliable MM-PB(GB)SA binding free energy calculations of HIV-1 protease inhibitors. J Comput Chem 29:673–685. doi:10.1002/jcc.20821. 17849388

[95] Zhai J, Dong X, Yan F, Guo H, Yang J. 2022. Oleandrin: a systematic review of its natural sources, structural properties, detection methods, pharmacokinetics, and toxicology. Front Pharmacol 13:822726. doi:10.3389/fphar.2022.822726. 35273501 PMC8902680

[96] Mustafa O, A UT, Cem A. 2004. Antibacterial activity of some actinomycetes isolated from farming soils of Turkey. Afr J Biotechnol 3:441–446. doi:10.5897/AJB2004.000-2087.

[97] Talukdar M, Bordoloi M, Dutta PP, Saikia S, Kolita B, Talukdar S, Nath S, Yadav A, Saikia R, Jha DK, Bora TC. 2016. Structure elucidation and biological activity of antibacterial compound from *Micromonospora auratinigra*, a soil actinomycetes. J Appl Microbiol 121:973–987. doi:10.1111/jam.13233. 27406903

[98] Benli M, Yiğit N, Geven F, Güney K, Bingöl U. 2008. Antimicrobial activity of endemic *Crataegus tanacetifolia* (Lam.) Pers and observation of the inhibition effect on bacterial cells. Cell Biochem Funct 26:844–851. doi:10.1002/cbf.1515. 18946875

[99] Sudha S, Masilamani Selvam M. 2011. *Streptomyces cavourensis* sp. SU 3 nov., a novel marine *Streptomyces* isolated from a sea shore sediment in Chennai. Advanced Biotech 2011:2–36.

[100] Shomura T, Yoshida J, Amano S, Kojima M, Inouye S, Niida T. 1979. Studies on actinomycetales producing antibiotics only on agar culture i. screening, taxonomy and morphology-productivity relationship of *Streptomyces halstedii*, strain SF-1993. J Antibiot (Tokyo) 32:427–435. doi:10.7164/antibiotics.32.427. 528390

[101] El-Gendy M, Shaaban M, Shaaban KA, El-Bondkly AM, Laatsch H. 2008. Essramycin: a first triazolopyrimidine antibiotic isolated from nature. J Antibiot (Tokyo) 61:149–157. doi:10.1038/ja.2008.124. 18503193

[102] Weisburg WG, Barns SM, Pelletier DA, Lane DJ. 1991. 16S ribosomal DNA amplification for phylogenetic study. J Bacteriol 173:697–703. doi:10.1128/jb.173.2.697-703.1991. 1987160 PMC207061

[103] Altschul SF, Gish W, Miller W, Myers EW, Lipman DJ. 1990. Basic local alignment search tool. J Mol Biol 215:403–410. doi:10.1016/S0022-2836(05)80360-2. 2231712

[104] Kim O-S, Cho Y-J, Lee K, Yoon S-H, Kim M, Na H, Park S-C, Jeon YS, Lee J-H, Yi H, Won S, Chun J. 2012. Introducing EzTaxon-e: a prokaryotic 16S rRNA gene sequence database with phylotypes that represent uncultured species. Int J Syst Evol Microbiol 62:716–721. doi:10.1099/ijs.0.038075-0. 22140171

[105] Thompson JD, Gibson TJ, Higgins DG. 2003. Multiple sequence alignment using ClustalW and ClustalX. CP Bioinformatics 00:2–3. doi:10.1002/0471250953.bi0203s00. 18792934

[106] Saitou N, Nei M. 1987. The neighbor-joining method: a new method for reconstructing phylogenetic trees. Mol Biol Evol 4:406–425. doi:10.1093/oxfordjournals.molbev.a040454. 3447015

[107] Kumar S, Stecher G, Li M, Knyaz C, Tamura K. 2018. MEGA X: Molecular 1323 Evolutionary Genetics Analysis across computing platforms. Mol Biol Evol 35:1547–1549. doi:10.1093/molbev/msy096. 29722887 PMC5967553

[108] Felsenstein J. 1985. Confidence limits on phylogenies: an approach using the bootstrap. Evolution 39:783–791. doi:10.1111/j.1558-5646.1985.tb00420.x. 28561359

[109] Ayuso-Sacido A, Genilloud O. 2005. New PCR primers for the screening of NRPS and PKS-I systems in actinomycetes: detection and distribution of these biosynthetic gene sequences in major taxonomic groups. Microb Ecol 49:10–24. doi:10.1007/s00248-004-0249-6. 15614464

[110] Williamson N, Brian P, Wellington EM. 2000. Molecular detection of bacterial and *streptomycete* chitinases in the environment. Antonie Van Leeuwenhoek 78:315–321. 11386354 10.1023/a:1010225909148

[111] Dutta J, Thakur D. 2021. Diversity of culturable bacteria endowed with antifungal metabolites biosynthetic characteristics associated with tea rhizosphere soil of Assam, India. BMC Microbiol 21:1–3. doi:10.1186/s12866-021-02278-z. 34275448 PMC8286567

[112] Khater S, Gupta M, Agrawal P, Sain N, Prava J, Gupta P, Grover M, Kumar N, Mohanty D. 2017. SBSPKSv2: structure-based sequence analysis of polyketide synthases and non-ribosomal peptide synthetases. Nucleic Acids Res 45:W72–W79. doi:10.1093/nar/gkx344. 28460065 PMC5570206

[113] Dutta B, Deska J, Bandopadhyay R, Shamekh S. 2021. In silico characterization of bacterial chitinase: illuminating its relationship with archaeal and eukaryotic cousins. J Genet Eng Biotechnol 19:19–11. doi:10.1186/s43141-021-00121-6. 33495874 PMC7835276

[114] Elshikh M, Ahmed S, Funston S, Dunlop P, McGaw M, Marchant R, Banat IM. 2016. Resazurin-based 96-well plate microdilution method for the determination of minimum inhibitory concentration of biosurfactants. Biotechnol Lett 38:1015–1019. doi:10.1007/s10529-016-2079-2. 26969604 PMC4853446

[115] Kamiloglu S, Sari G, Ozdal T, Capanoglu E. 2020. Guidelines for cell viability assays. Food Frontiers 1:332–349. doi:10.1002/fft2.44.

[116] Stoddart MJ. 2011. Cell viability assays: introduction. Mammalian Cell Viability 2011:1–6. doi:10.1007/978-1-61779-108-6_1. 21468961

